# The evolution of pandemic influenza: evidence from India, 1918–19

**DOI:** 10.1186/1471-2334-14-510

**Published:** 2014-09-19

**Authors:** Siddharth Chandra, Eva Kassens-Noor

**Affiliations:** Asian Studies Center, Michigan State University, 301 International Center, East Lansing, MI 48824 USA; School of Planning Design and Construction and Global Urban Studies Program, Michigan State University, East Lansing, MI 48824 USA

**Keywords:** 1918 influenza pandemic, Spanish Influenza, Influenza, Pandemic, Mortality, India, Epidemic velocity, Climatic factors, Immunity

## Abstract

**Background:**

The 1918–19 ‘Spanish’ Influenza was the most devastating pandemic in recent history, with estimates of global mortality ranging from 20 to 50 million. The focal point of the pandemic was India, with an estimated death toll of between 10 and 20 million. We will characterize the pattern of spread, mortality, and evolution of the 1918 influenza across India using spatial or temporal data.

**Methods:**

This study estimates weekly deaths in 213 districts from nine provinces in India. We compute statistical measures of the severity, speed, and duration of the virulent autumn wave of the disease as it evolved and diffused throughout India. These estimates create a clear picture of the spread of the pandemic across India.

**Results:**

Analysis of the timing and mortality patterns of the disease reveals a striking pattern of speed deceleration, reduction in peak-week mortality, a prolonging of the epidemic wave, and a decrease in overall virulence of the pandemic over time.

**Conclusions:**

The findings are consistent with a variety of possible causes, including the changing nature of the dominant viral strain and the timing and severity of the monsoon. The results significantly advance our knowledge of this devastating pandemic at its global focal point.

**Electronic supplementary material:**

The online version of this article (doi:10.1186/1471-2334-14-510) contains supplementary material, which is available to authorized users.

## Background

Recent influenza outbreaks around the world have generated renewed interest in the study of the spread and properties of pandemic diseases with a view to mitigating their impacts [1–6]. The so-called Spanish Influenza of 1918 was the most devastating in history, with a global death toll of between 20 and 50 million [7–10]. Caused by a member of the H1N1 family of influenza viruses, the first wave of the disease emerged in the early months of 1918. In the autumn of 1918, a second and severe wave of the disease swept across the globe, leaving no major inhabited region untouched [7, 8, 11–19]. The focal point of the epidemic in terms of mortality was India, with an estimated death toll range of 10–20 million, and a point estimate of population loss of 13.8 million for the British-controlled provinces [8, 20, 21]. Surprisingly, no modern study exists that comprehensively characterizes the spatial dynamics of the 1918 influenza in India even though Mills [22] highlighted its importance through his description of its effects on India. The aim of this study is to use a comprehensive dataset to identify important features of the 1918–19 influenza pandemic in India that are of interest for the understanding of influenza epidemics. Striking findings include a reduction in peak-week mortality, a deceleration in velocity, a prolonging of the epidemic wave, and an overall reduction in virulence of the Spanish Influenza as it spread across India.

Analyzing diffusion patterns of pandemics is difficult, as it depends upon various factors including place-specific public health responses, social interactions among people, travel patterns within cities and across countries, the natural and built environments, and characteristics of the pathogens themselves [5, 23, 24]. For the Spanish Influenza, Crosby [7] concluded that "the factors at work in the pandemic were so numerous … that very few generalities can be drawn." One such generality, also observed in India, was the phenomenon of two distinct epidemic waves, a mild one in the spring or summer of 1918, and a second and much more lethal one in the autumn or winter [8]. Some countries experienced a third wave in 1919 or 1920 [11]. In an early attempt to characterize the spread of the pandemic, Pyle [12] presented diffusion patterns of the disease alongside those of other influenza epidemics of the 20th century. For England, Mexico and Peru, Chowell et al. [14, 25, 26] identified spread patterns, estimating mortality and transmissibility rates and distinguishing between rural and urban areas. Across Europe, several researchers including Ansart et al. [27] and Erkoreka [28] estimated the mortality burden of the 1918 Pandemic. In addition, early in the second wave, long distance infections were the predominant mode of transmission, with local infections becoming more frequent as the epidemic became widespread [29].

In India, the second wave originated in Bombay in September 1918, simultaneously spreading north and south, and reaching Sri Lanka and the northern Indian provinces in October 1918 [11, 30]. The pandemic is believed to have originated from influenza-infected World War I troops returning home [7, 11, 12, 31, 32]. The disease was passed on to and spread amongst civilian populations in different regions. Transportation systems aided the spread of the disease. In the case of India, the Sanitary Commissioner in his report [33] noted that "The railway played a prominent part as was inevitable" (p.61; see also Pyle and Patterson [12]).

Taken from the Sanitary Commissioner’s Report for 1918, Figure 1 below shows the weekly death rate in three key Indian cities, Bombay, Madras, and Calcutta. Bombay, the westernmost of the three cities, was thought to be the entry point of the virus into India [34]. Of the three cities, it was the first to experience the first and second waves of the epidemic, with an autumn wave that was shorter and more pronounced than those for the other cities [22]. Madras, to the southeast of Bombay, experienced the wave slightly later and in a less (albeit still) pronounced manner, and Calcutta, the easternmost of the three cities, experienced a prolonged but altogether less prominent second wave. A summary of the progress of the pandemic across five major British Indian cities, based on the description provided in the Sanitary Report [33] (p.58-59), suggests a relationship between location, timing, duration, and severity of the pandemic. Specifically, Bombay, on the west coast of India, experienced the beginning of the wave in the week of September 7–14, with a peak in the week of September 29 to October 5, for a start-to-peak duration of three weeks [33]. The provincial death rate in the Bombay Presidency was a relatively high 54.9 people per thousand inhabitants [33]. The corresponding figures for other major cities included, for Madras (on the south eastern coast of India), a start in the week of September 14–21, a peak during the week of October 13–19, a start-to-peak duration of four weeks, and a death rate of 16.7 [33]. For Calcutta, in eastern India, the figures included a start during the week of September 14–21, a maximum death rate during the week of November 16–23, a start-to-peak duration of eight weeks, and a provincial death rate of 8.5 [33]. A variety of methods and data will be used to test the extent to which this pattern is systematic and is replicated across the entirety of British India.

**Figure 1:**
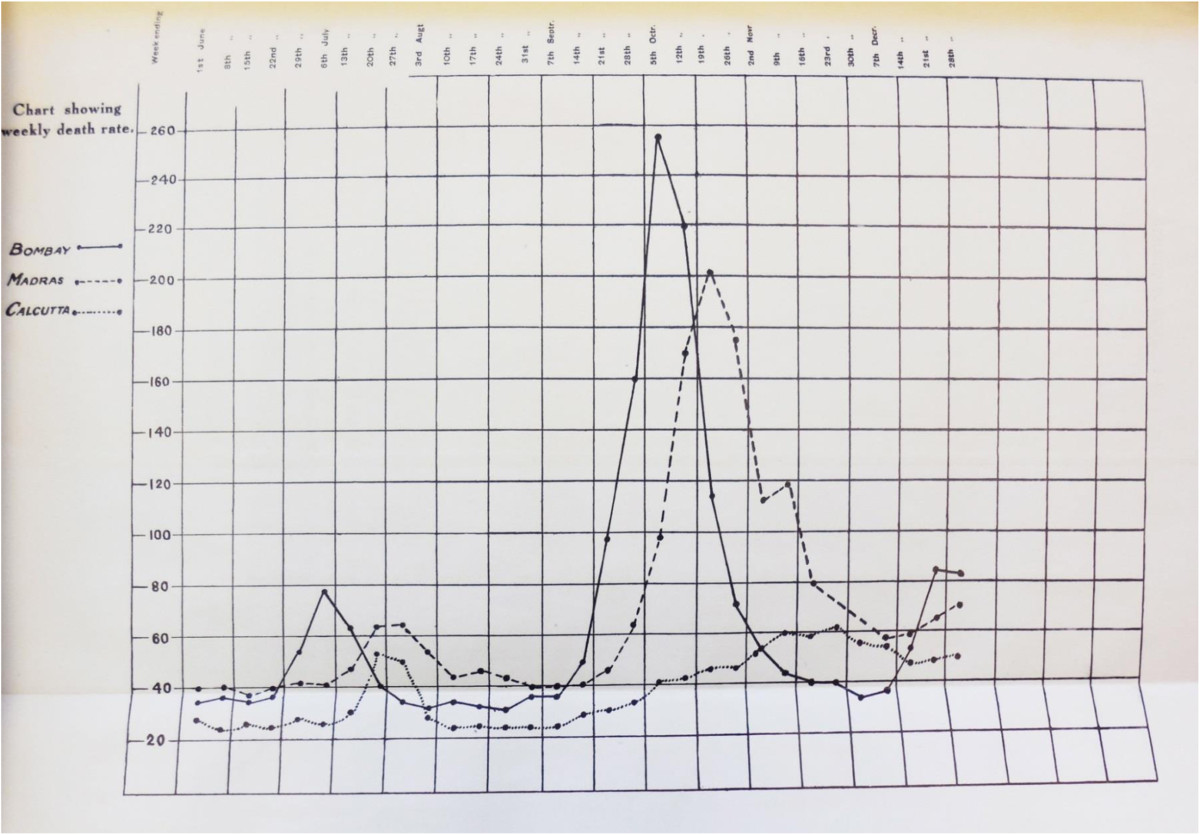
**Weekly Death rates in Bombay, Madras, and Calcutta, June-December, 1918.**

## Methods

The statistical data are obtained from the sanitary reports of the provinces or presidencies of Assam, Bengal, Bihar and Orissa, Bombay, Central Provinces, Madras, Northwest Frontier Province, Punjab, and United Provinces for the five-year period from 1916 to 1920 [33, 34]. The data were copied from these public reports and entered into a database for analysis. This places 1918, the peak year of the pandemic in India, in the temporal center of the dataset. The nine aforementioned provinces formed the major administrative units of India that were directly ruled by the British. The boundary files for district maps were created by modifying modern-day district boundaries using the information in the census atlas of India [35] to arrive at the colonial district boundaries in 1918.

The Sanitary Commissioner [33] (p. 64) reported four syndromes associated with the pandemic influenza, "(a) febrile, (b) bronchial, (c) intestinal, [and] (d) meningeal." The provincial sanitary reports contain monthly counts of deaths from "fevers" for 213 districts from 1916–1920. While "fevers" encompass a variety of conditions, the vast majority of the deaths from the pandemic were recorded under this heading, with the 11,134,441 reported fever deaths far exceeding the decennial mean of 4,308,356 [33]. While data were also reported on mortality from respiratory diseases, we chose to use the "fevers" data because pneumonia and bronchopneumonia were often complications that followed an attack of influenza rather than occurring, like fevers, when the disease itself peaked. Therefore, fevers are a more accurate indicator of when the influenza was occurring in a particular locale. Second, and equally importantly, the "fevers" heading shows a much more dramatic spike in mortality during the pandemic than the "respiratory diseases" heading. Data on influenza cases, while desirable, are unavailable, hence the focus of this paper is on the information conveyed by the mortality statistics instead. These data show a steep spike in fever mortality in late 1918, reflecting the autumn wave of the pandemic. Therefore, while we recognize that the "fever" heading may also have contained deaths from other fever-causing diseases, most importantly seasonal malaria, we focus on the "fevers" data category for this analysis after adjusting it for the presence of seasonal fever deaths from all causes [20]. As is demonstrated below, the use of the "fevers" data does not pose a serious problem for the estimation of the *timing* of peak influenza-attributable mortality in the districts because of the sheer number of deaths from the pandemic. For this reason, we use seasonally adjusted deaths from "fevers" to capture the temporal and spatial characteristics of the pandemic.

**Figure 2:**
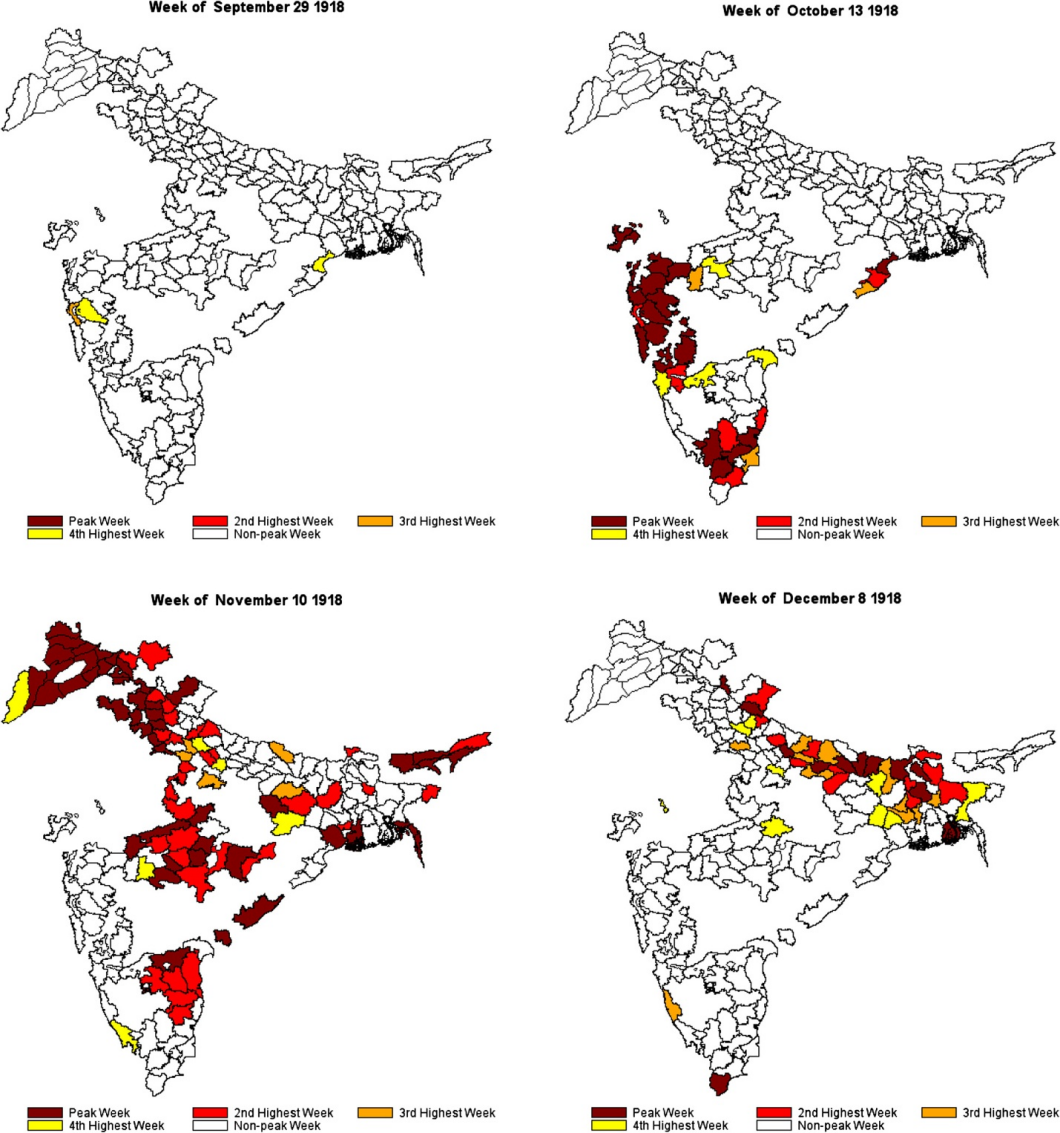
**Districts with Peak Mortality During the Influenza Pandemic of 1918–1919.**

The analyses proceeded as follows. First, the monthly data were seasonally adjusted using the X11 algorithm in the SAS software to eliminate those deaths from fevers that could be attributed to seasonal fever-causing diseases such as malaria. This adjustment, using an additive model, was conducted on a district-by-district basis to account for the variation in climate and seasonal variability of disease-related variables such as rainfall and temperature across India. Because of the large number of districts, rather than manually select models for each district, we used the TRAMO method of model selection [36]. The maximal order for nonseasonal differencing was 2 and that for seasonal differencing was 1. We estimated two sets of data, one including all the data, and the second controlling for outliers in view of the extreme conditions during the pandemic. In the second step, the seasonally adjusted monthly data, reflecting excess fever mortality by month across districts, were interpolated using the PROC EXPAND procedure in SAS to create weekly time series for the five-year period, consisting of 262 weekly estimates, for each district [37]. Third, for each district, we created a ranking of the 262 weekly mortality estimates and selected the four weeks in this 262-week time-span with the highest mortality as indicative of the peak of the virulent autumn wave of the pandemic. The utility of the "fevers" statistics as indicators of the timing of the epidemic was confirmed by the timing of these peak weeks, which occurred in the autumn of 1918, and the clear pattern by which they demonstrate that the disease originated in Bombay and spread out across India from there (see Figure 2 and the Additional file 1: Video clip in the "Results" section below).

**Figure 3:**
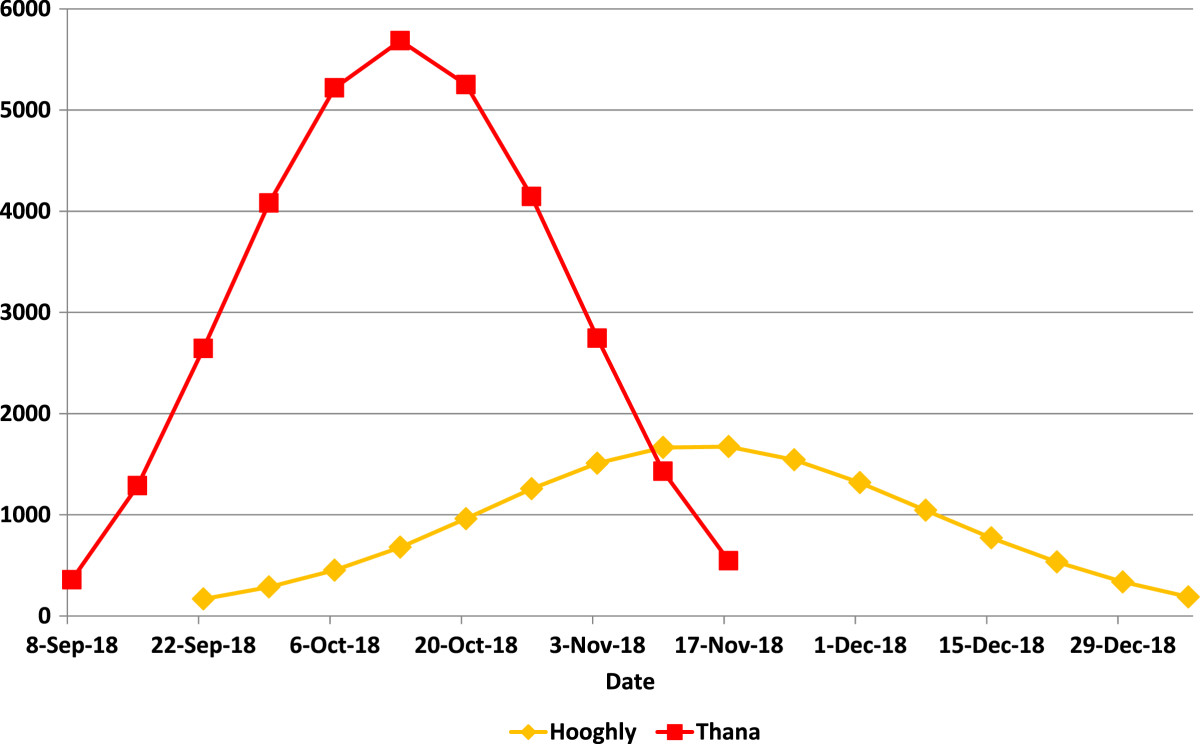
**Autumn Pandemic Waves in Thana and Hooghly Districts (Seasonally Adjusted Weekly Excess "Fevers" Mortality).**

Next, having established the usefulness of the fevers data for the analysis of temporal characteristics of the influenza pandemic as it spread across India, we computed a variety of characteristic measures of the geographic and temporal properties of the pandemic in each district with a view to understanding its evolution. For this purpose, we defined the epidemic phase of the disease based on the CDC’s definition [38]: "Epidemic refers to an increase, often sudden, in the number of cases of a disease above what is normally expected in that population in that area." For each district, therefore, we used a threshold death count of at least one standard deviation above the mean number of excess deaths between 1916 and 1920 to indicate the presence of an epidemic wave. We also conducted sensitivity analyses of the results using two standard deviations as an alternate cut-off in identifying the epidemic wave, and found that the results were very similar. The contrast between the estimated autumn epidemic waves for the districts of Thana (near Bombay) and Hooghly (near Calcutta) in Figure 3 mirrors the contrast between the reported waves in Bombay and Calcutta in Figure 1, suggesting a systematic geographic pattern.

To study patterns of propagation of influenza across the districts, we used the data on the wave to compute four sets of two measures each to capture timing, length, severity, and shape of the wave. The timing of the wave was measured using (i) the date on which the wave peaked (*T*_max_) and (ii) the midpoint in time of the wave (i.e., halfway between the start- (*T*_start_) and end-dates (*T*_end_) of the wave, or $\left(\frac{T_{\text{end}}-T_{\text{start}}}{2}\right)$).

The length or duration of the wave was measured as (i) the number of days between the start and the end of the wave (*T*_end_ - *T*_start_) and, following Trevelyan, Smallman-Raynor and Cliff [39] and based on methods developed and reviewed by Cliff et. al [40] and Cliff and Haggett [41], (ii) as the mean time to mortality, or epidemic velocity. Here velocity is defined as the mean number of days to death (data on infections are not available, hence we used data on deaths to compute a modified indicator of velocity), calculated as

$$\bar{t}=\frac{1}{N}\sum_{t=T_{\mathrm{start}}}^{T_{\mathrm{end}}}tx_{t}$$

where $\bar{t}$ is the mean time to death, *t* is a point in time measured in days from the start of the epidemic, *x*_*t*_ is the number of excess deaths at time *t*, *T*_end_ is the end date of the epidemic as defined above [42], and $N=\sum_{T_{\mathrm{start}}}^{T_{\mathrm{end}}}x_{t}\text{.}$ The severity of the wave was measured using (i) the total number of deaths at the peak of the epidemic $\left(x_{T_{\text{max}}}\right)$ and (ii) the number of deaths at the peak of the epidemic normalized by the standard deviation of deaths for the district $\left(\frac{x_{T_{\text{max}}}}{\sigma_{N}}\right)\text{.}$ Finally, measures of the shape of the wave included (i) its variance and (ii) a measure of its skewness, defined in Cliff and Haggett [41] as

$$\sqrt{b_{1}}=\frac{m_{3}}{m_{2}^{\frac{3}{2}}}$$

where

$$m_{r}=\sum_{t=T_{\mathrm{start}}}^{T_{\mathrm{end}}}\left\{{\left(t-\bar{t}\right)}^{r}x_{t}\right\}/N$$

**Figure 4:**
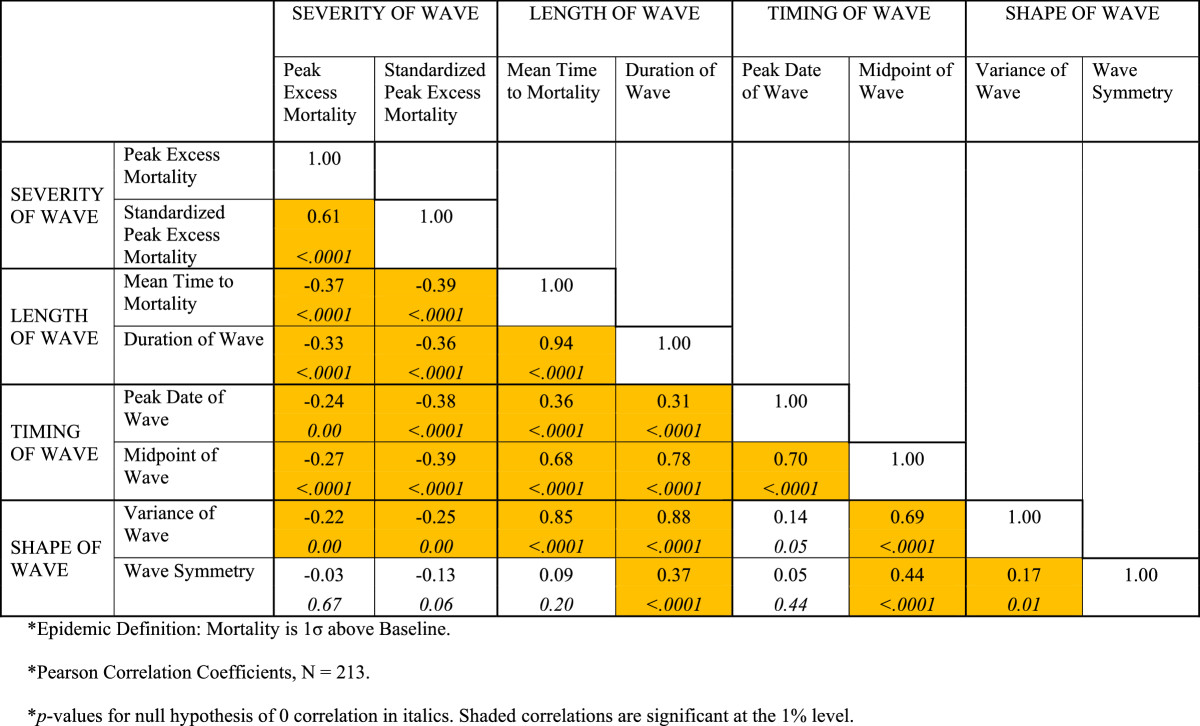
Correlations between variables capturing the dynamics of pandemic influenza in India, 1918-19*.

Summary statistics for the eight measures defined above are provided in Table 1.Associations between these groups of variables were computed using Pearson correlation coefficients (Figure 4 below).

**Table 1:** Summary statistics on dynamics of the autumn wave* of the 1918 influenza pandemic ( ***n*** **= 213 districts)**

CATEGORY OF VARIABLE	Variable	Mean and Standard Deviation
SEVERITY OF WAVE	Peak Excess Mortality	4223.00
		(3083)
	Standardized Peak Excess Mortality	20.76
		(10.57)
LENGTH OF WAVE	Mean Time to Mortality (Days)	51.93
		(35.00)
	Duration of Wave (Weeks)	17.85
		(12.74)
TIMING OF WAVE	Peak Date of Wave	Nov. 16, 1918
		(35.03)
	Midpoint of Wave	Dec. 03, 1918
		(56.75)
SHAPE OF WAVE	Variance of Wave	1399.00
		(3708.00)
	Wave Symmetry	0.25
		(0.50)

*The wave is defined as being current when deaths exceed 1σ over the baseline.

## Results

**Figure 5:**
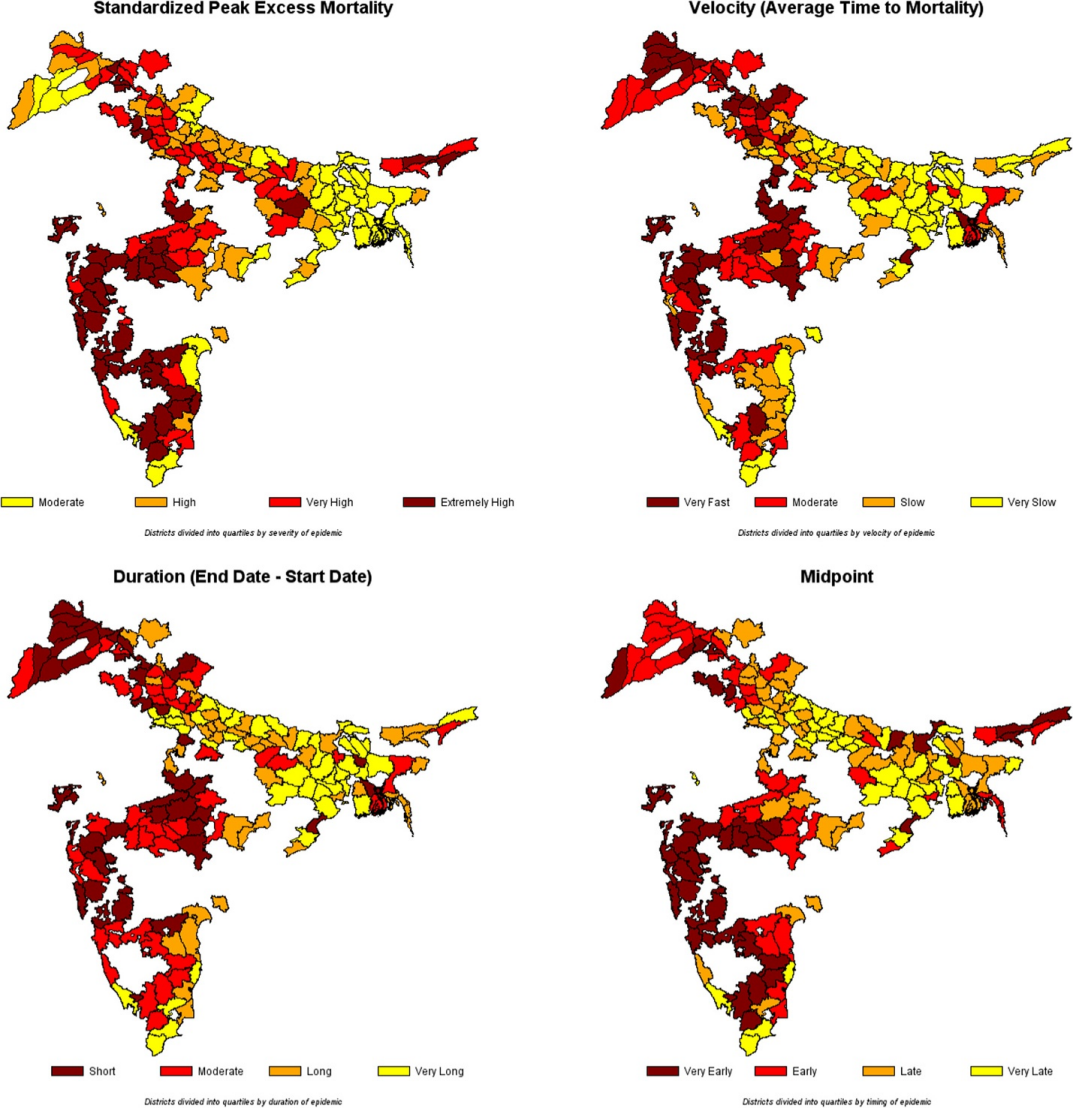
**Severity, Velocity, Duration, and Timing of the Fall Wave (1σ) of the Influenza Pandemic of 1918–1919.**

Using the weekly rankings described above, Figure 2 illustrates the progress of the deadly autumn wave through India. The wave originated in and radiated from Bombay on the west coast, and is suspected to have arrived in India on a troop ship carrying soldiers home from the First World War in Europe [33]. Different regions of India experienced successive episodes of peak mortality. The week of October 13, 1918 shows the early regional peaks in the western province of Bombay, the south-eastern province of Madras, and a small area on the eastern coast of India near the important Hindu pilgrimage site of Puri. Four weeks later, the epidemic peaked in the Central Provinces and the northwestern province of Punjab (week of November 10). Finally, another four weeks later, the epidemic peaked in the northern and eastern parts of the United Provinces, Bihar, and Bengal (week of December 8). This last set of peaks appears more scattered and sporadic than the more synchronous peaks seen in Bombay, Madras, the Central Provinces, and Punjab. The attached clip shows the weekly progression of the pandemic across India between September 1, 1918 and January 19, 1919 and the unmistakable wave-like manner in which the pandemic moved across India (Additional file 1).Figure 5 illustrates the districts arranged by quartile on the basis of four important characteristics of the pandemic --- timing, velocity, duration, and severity. The four maps are striking in that they demonstrate that, over time, (a) the severity of the epidemic diminished, (b) the velocity (average time to death) of the wave slowed down, (c) the wave grew longer in duration, and (d) the eastern portions of India were the last to experience the pandemic. In terms of severity, Bombay, the Central Provinces, and parts of Madras were hardest hit. The highest velocity (lowest mean time to mortality) districts appear to be concentrated in Bombay, the Central Provinces, and Punjab as are the shortest duration districts. The earliest midpoint districts, likewise, are concentrated in Bombay, the Central Provinces, and parts of Madras and Punjab.Figure 4 reports correlations for the variables, defined above, that capture the severity, velocity, duration, and timing of the pandemic. The first interesting feature of Figure 4 is the high degree of correlation between measures within each category (except for the shape of wave category, for which each measure (variance and skewness) captures a qualitatively different phenomenon). This suggests that the two severity measures capture the same phenomenon, as do the two length measures and the two timing measures.The correlations are consistent with the patterns observed in Figure 4. First, as the epidemic progressed throughout India, the severity of the wave diminished, as evidenced by the negative correlations between severity and timing. In addition, over time, the length of the wave \increased, as demonstrated by the positive correlations between the length of wave and timing of wave measures. Third, as the epidemic progressed, the shape of the wave became more skewed in the positive direction, suggesting a more rapid rise in the initial days of the epidemic and a longer trailing-off phenomenon. The severity of the wave was also negatively correlated with the length and variance of the wave, suggesting that waves that lasted longer tended to have lower peak mortality weeks. Since severity was also negatively associated with the timing of the wave, a phenomenon that is reinforced with the death rates reported by the Sanitary Commissioner (see above), these correlations paint an intriguing overall picture of the evolving autumn pandemic wave as it rolled across India.

## Discussion

The diminishing virulence and velocity of the epidemic wave and its lengthening as it progressed through India raises the important epidemiological question of why. Coupled with the observation that the spread in the northern portion of India was less spatially uniform than the spread earlier and elsewhere (Figures 2, 5, and the clip in the Additional file 1: Video clip), a number of possible explanations come to mind. Key among them is an important theme in the theory of evolution of epidemics --- competition among strains of a rapidly evolving virus can produce an equilibrium in which the predominant strain is less virulent and slower to travel than the strain that predominated at the onset of the epidemic [43–45].

A second key explanation could be weather. Recent studies suggest that absolute humidity constrains both influenza virus survival and transmission efficiency [46, 47]. The crucial summer monsoon rains were described as follows by the Sanitary Commissioner [33] (p.47):

"(iii) The monsoon rains (June to September) began earlier than usual, but were very weak over nearly the whole country outside of Burma and northeast India. The deficiency in the seasonal rainfall was as much as 81 per cent. in Sind, 75 per cent. in Rajputana West, 70 per cent. in Baluchistan, 63 per cent. in Guzerat, and about 50 per cent. in the United Provinces West, the Punjab, Rajputana East, Central India East, Berar, the Konkan, the Bombay Deccan, Mysore, and Malabar."

**Figure 6:**
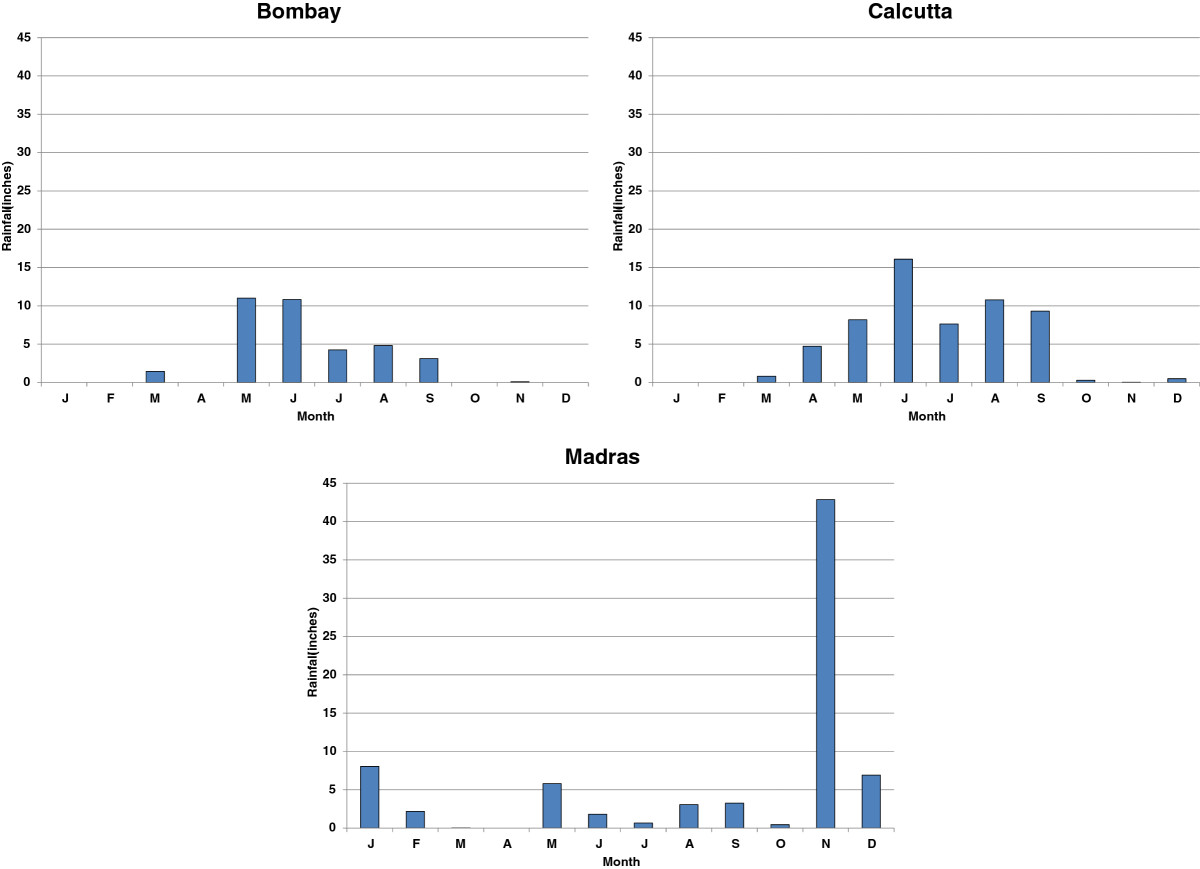
**Monthly Rainfall in Bombay, Calcutta, and Madras in 1918.**

Note the exclusion of Bengal and Bihar in the eastern part of India, from this list --- these were among the regions that were least severely affected by the pandemic. The monthly rainfall data during 1918 (Figure 6) in conjunction with the timing and severity data in Figure 4 are consistent with this explanation. In Bombay, where the "rainfall was very defective" [33] and the summer monsoon ended early, the survival and transmission of the virus may have been spurred during the autumn. In Madras, which experiences a winter monsoon, the autumn wave had to contend with an abnormally wet November, which may have stopped the pandemic. Calcutta, which had a wetter and longer summer monsoon than Bombay, may have been spared the virulence of the pandemic because of higher humidity. Indeed, the Sanitary Commissioner noted on several occasions in his report that the low mortality rate along the coast lines was striking [33], lending further support to this humidity hypothesis.

The findings of this paper, while not confirmatory, are broadly consistent with both notions, the theory of evolution of viral pathogens in an epidemic and the association with weather patterns. Given the complexity of epidemics, however, a number of additional factors may have been at play. These include knowledge of the pandemic that may have reached the eastern part of the country via conferences held on the topic [34], allowing simple social distancing measures to be enacted [6, 48] and early detection through influenza awareness of inspectors and doctors [33], thereby lowering virulence in the later stages of the epidemic. Another possible interpretation is that populations in the north and east of India may have acquired a higher degree of immunity, or cross-protection from the first wave of the influenza and therefore were not as severely affected by the second wave as their southern and western counterparts [49–51]. The deceleration of the epidemic as it radiated outward from Bombay may also have resulted from multiple influenza carriers being introduced at once into a susceptible population in Bombay, thereby spreading the virus faster, while fewer influenza carriers traveled across the country on account of the rapid and severe onset of the disease [33]. The spread from west to east may be explained by the virus being introduced through European troops. In particular, some British Indian troops were stationed in France during World War I, and the first returnees in India most likely disembarked in western India [52]. Finally, rurality may have contributed to the weakening of the pandemic’s spread [53].

## Conclusions

As the centenary of the 1918–1919 pandemic approaches, scholars are returning to its study to design appropriate pandemic control strategies [54–56]. By establishing a baseline picture of how the worst epidemiologic disaster of the modern era unfolded at its global focal point, it is hoped that this study will prompt future research that delves into the complex factors that created and shaped it spatially and temporally [8, 57, 58]. The lengthening and weakening of the pandemic wave as it swept across India also has broad implications for pandemic control strategies. The duration and intensity of pandemic control measures will need to be judiciously calibrated to the variable nature of any future pandemic wave. In scenarios resembling the 1918 pandemic as it unfolded in India, locations close to an entry point will have extremely short windows of time to deal with a virulent pathogen, placing emphasis on the emergency management of a short and severe wave of illness. While locations that are distant from the entry point will have longer windows of time to prepare for and deal with less lethal variants of the disease, their task will be prolonged by the more gradual build-up and subsidence of the epidemic wave.

### Ethical approval

Ethical approval was not required for the research conducted in this study.

## Electronic supplementary material

Additional file 1: Clip showing the weekly progression of the influenza pandemic across India between September 1, 1918 and January 19, 1919.(PPTX 523 KB)

Below are the links to the authors’ original submitted files for images.

Authors’ original file for figure 1

Authors’ original file for figure 2

Authors’ original file for figure 3

Authors’ original file for figure 4

Authors’ original file for figure 5

Authors’ original file for figure 6
