# Supplementary material for: The evolution of pandemic influenza: evidence from India, 1918–19
Source: BMC Infect Dis. 2014 Sep 19;14:510. doi: 10.1186/1471-2334-14-510 (PMC4262128; doi:10.1186/1471-2334-14-510)
Supplement: Supplementary file 1 — Additional file 1: Clip showing the weekly progression of the influenza pandemic across India between September 1, 1918 and January 19, 1919.(PPTX 523 KB) [file 12879_2014_3837_MOESM1_ESM.pptx]

## Slide 1
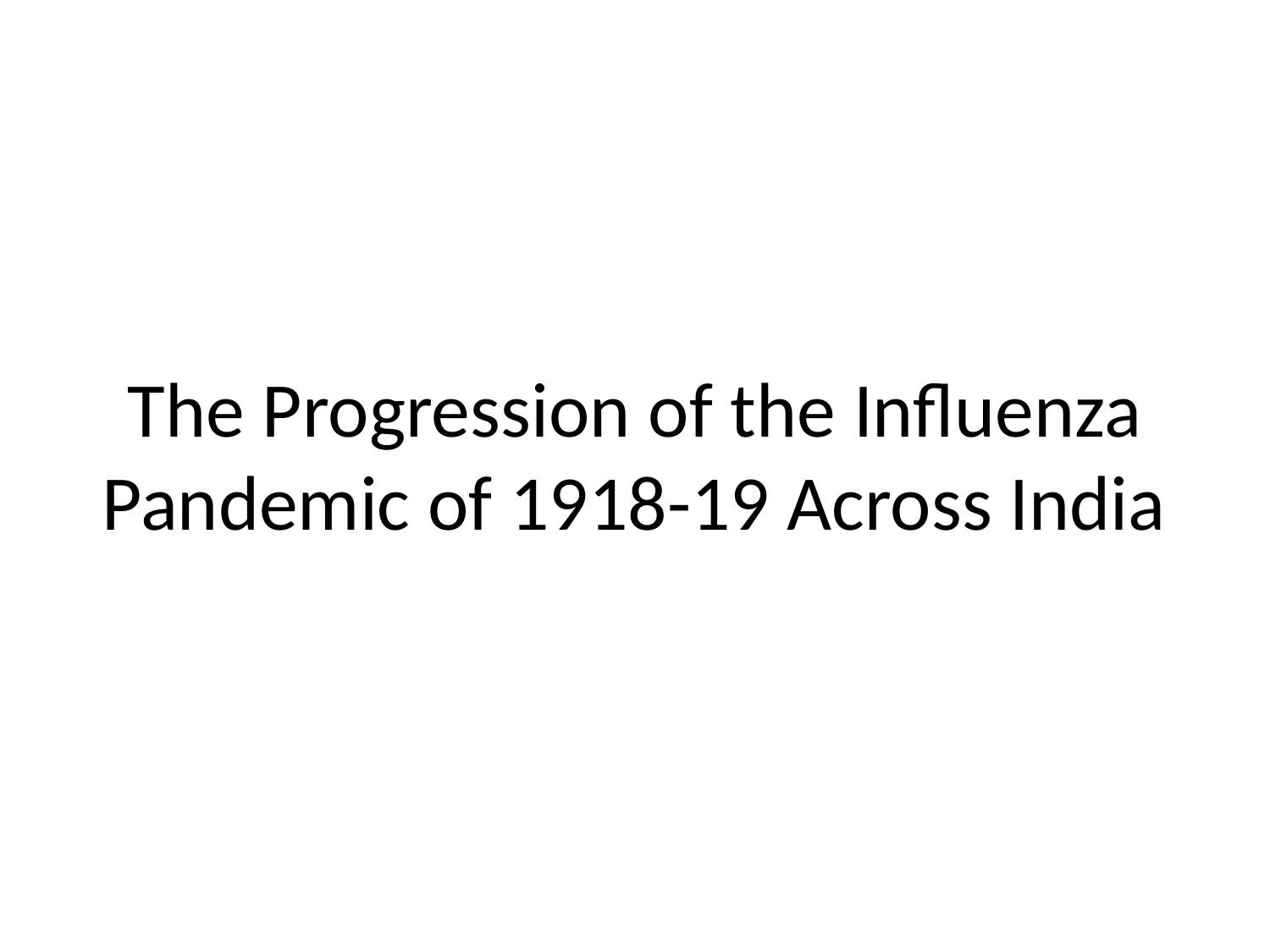

# The Progression of the Influenza Pandemic of 1918-19 Across India

## Slide 2
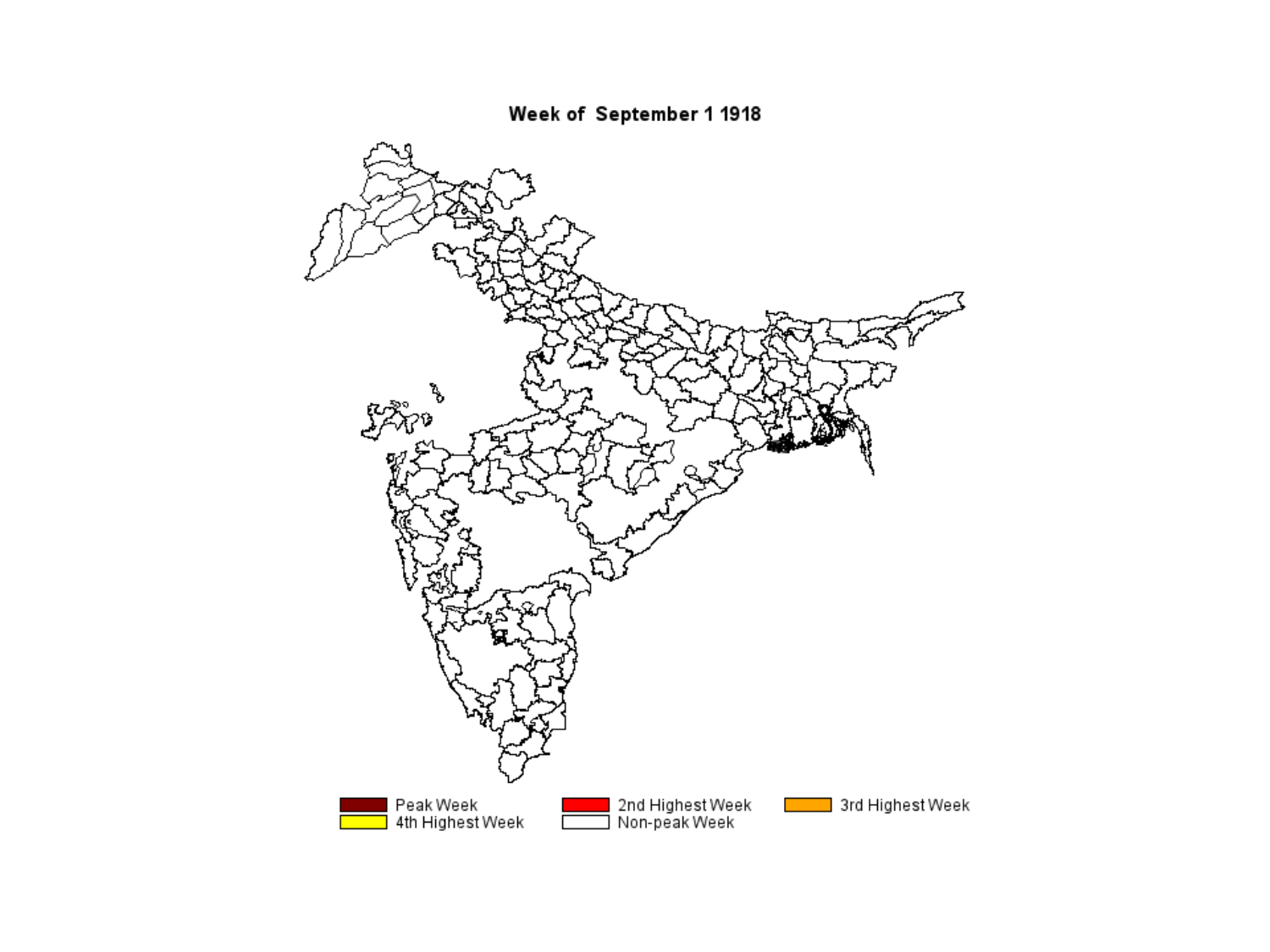

## Slide 3
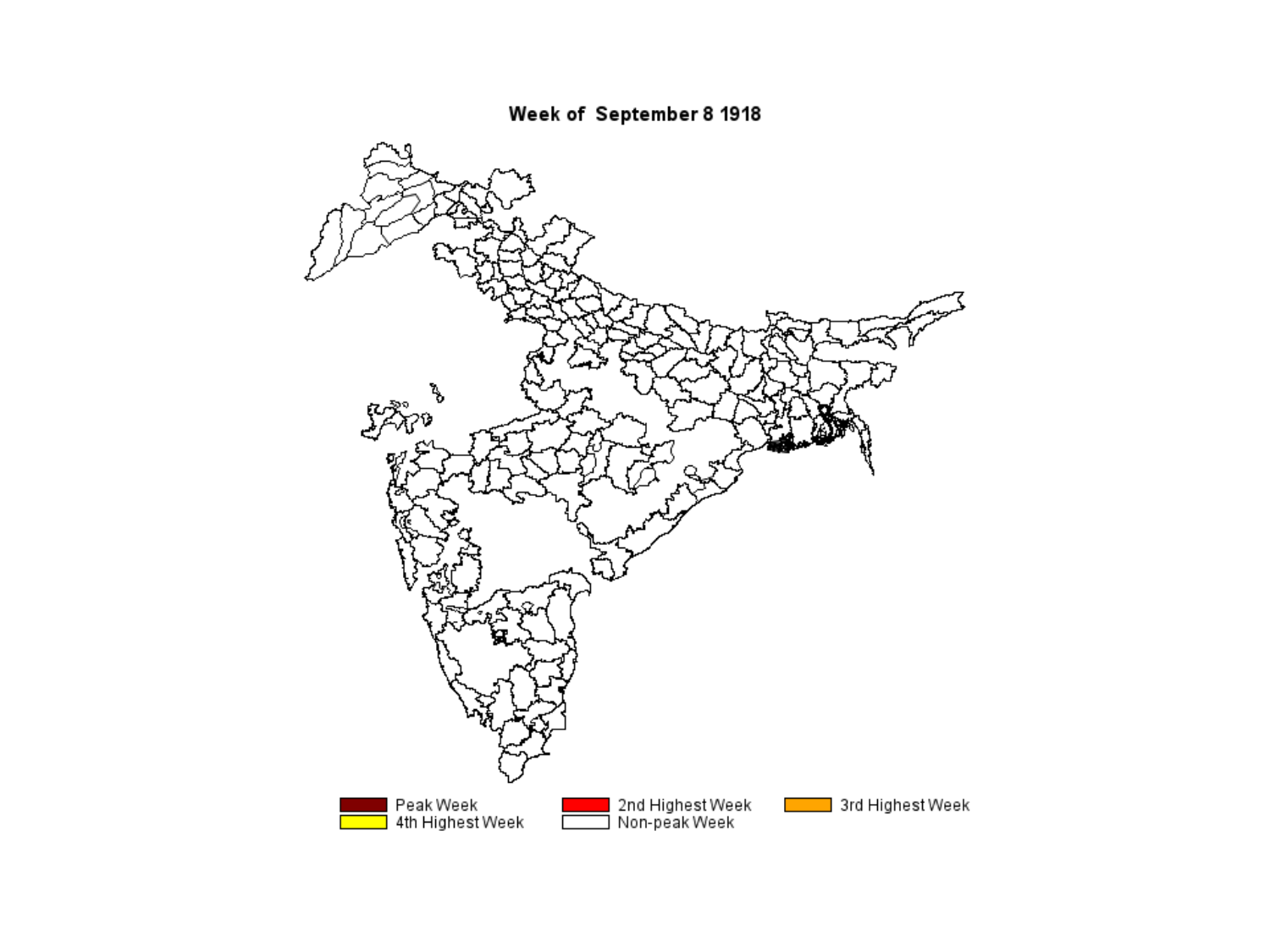

## Slide 4
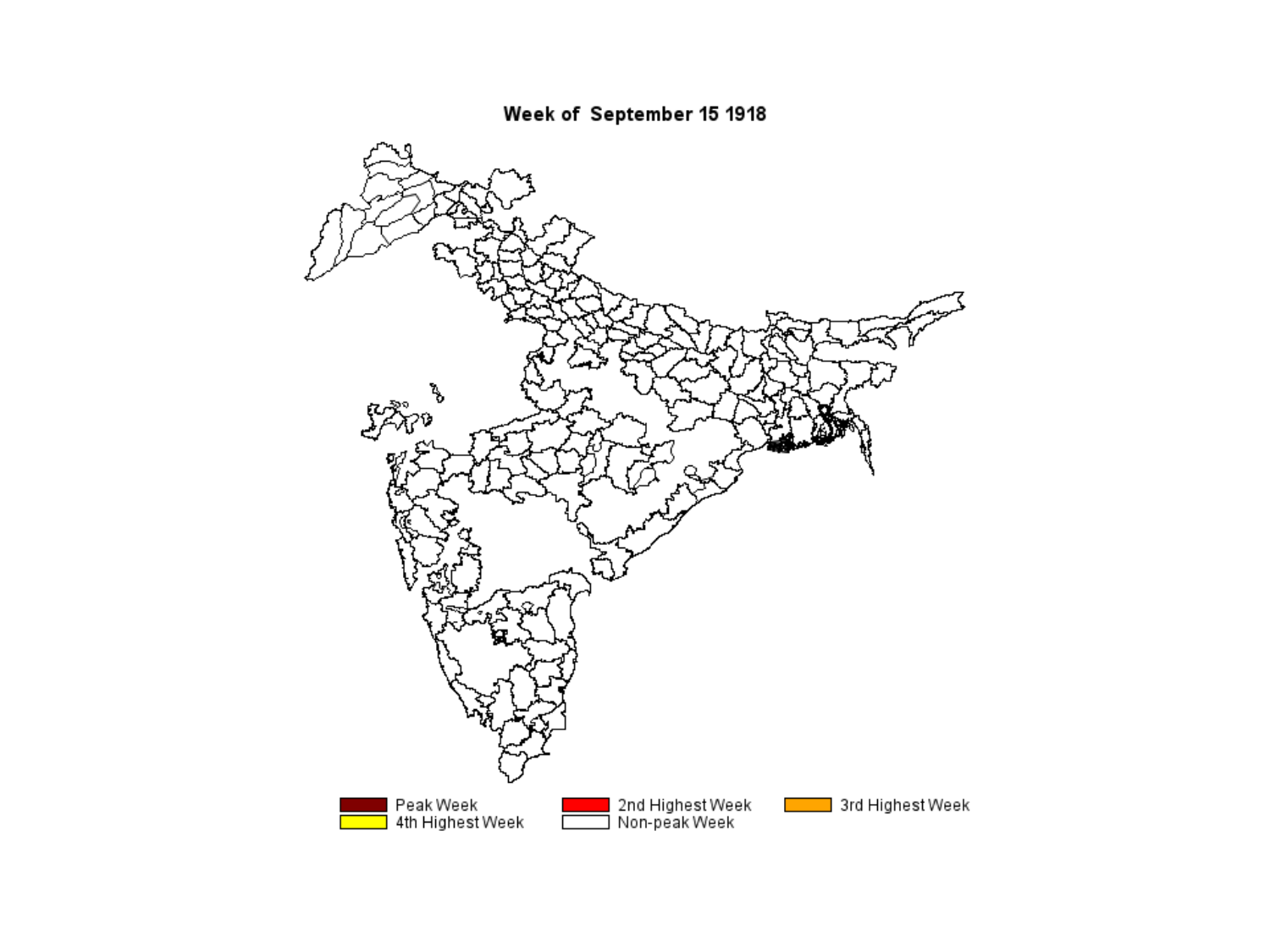

## Slide 5
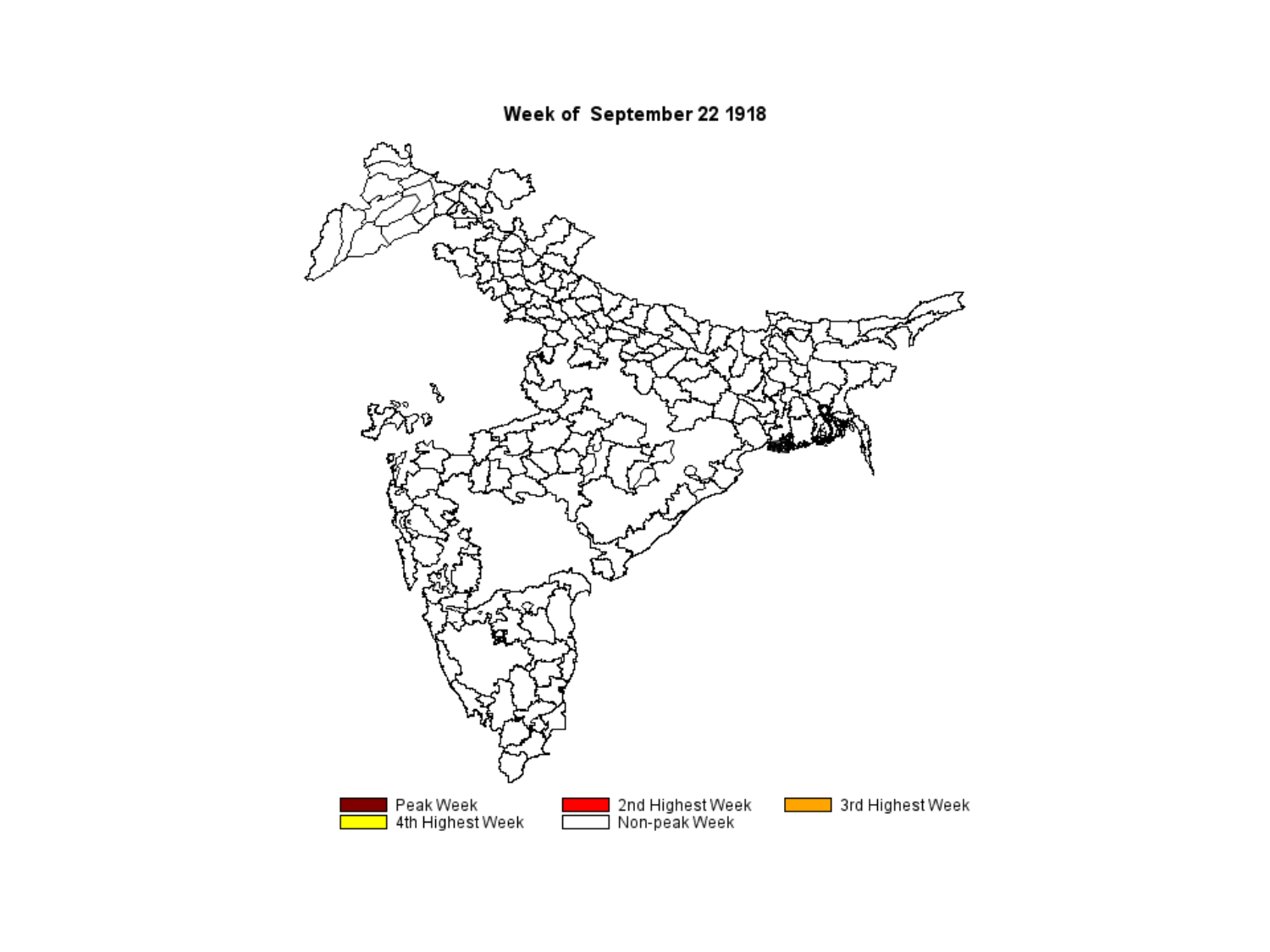

## Slide 6
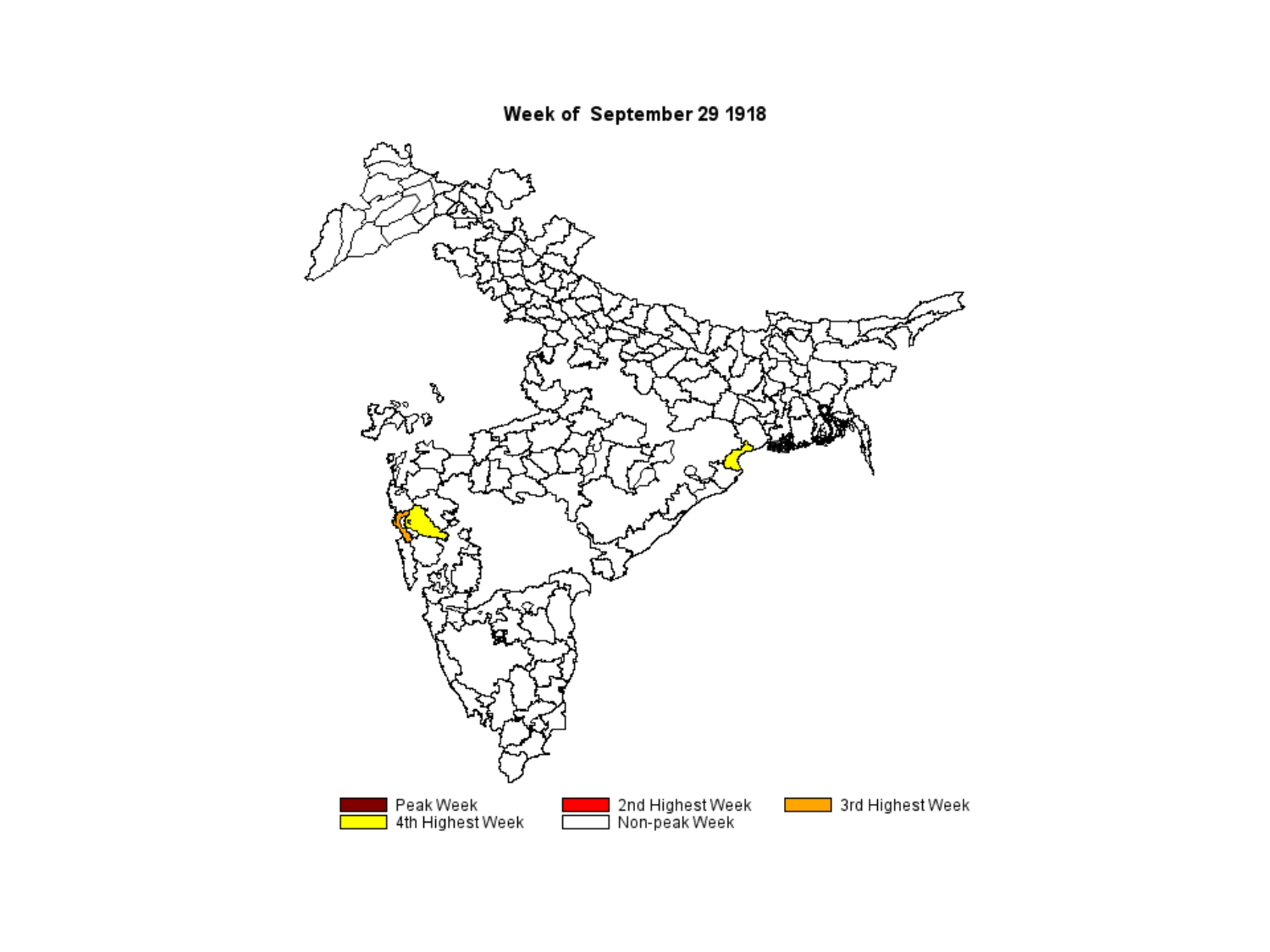

## Slide 7
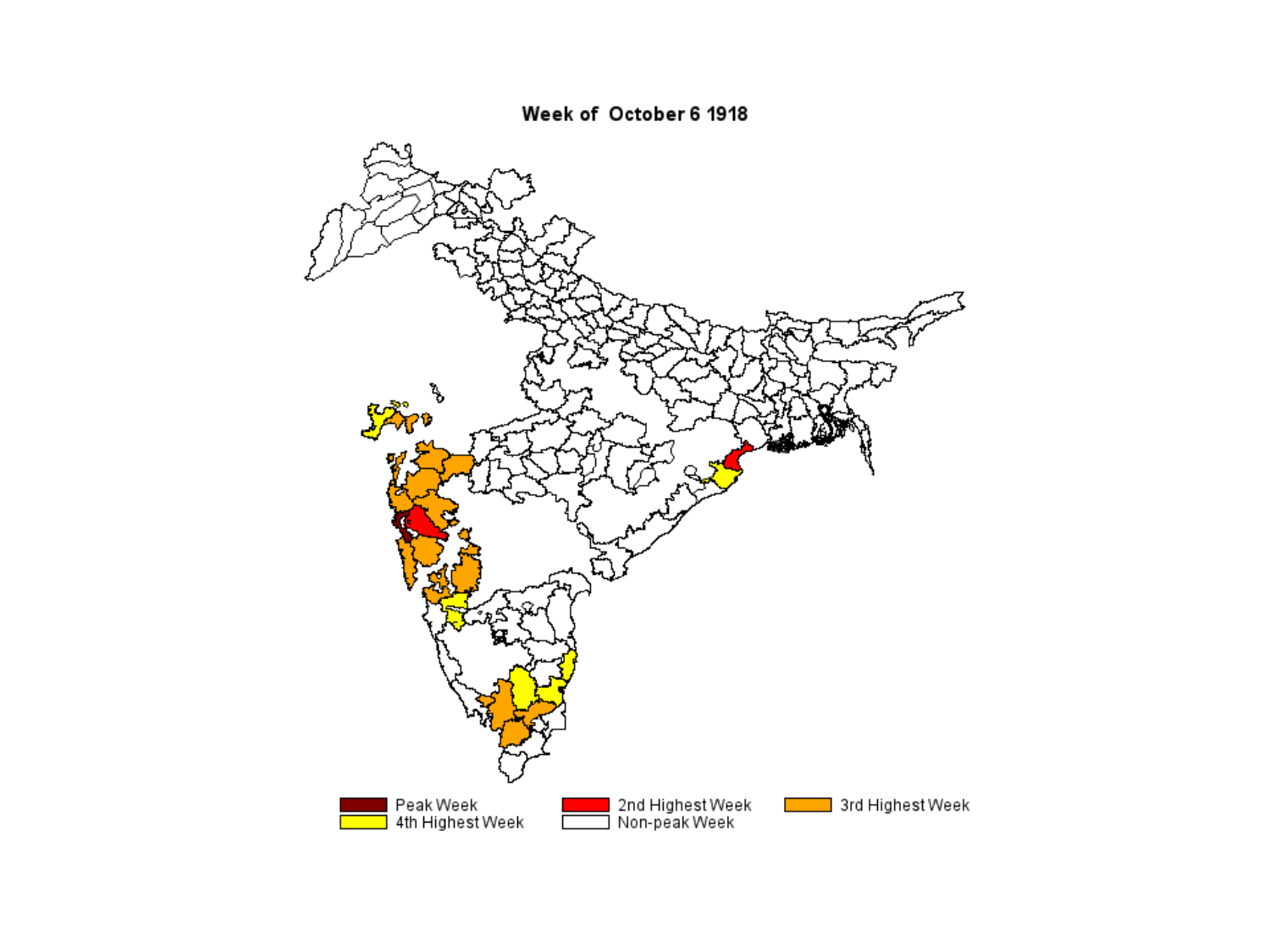

## Slide 8
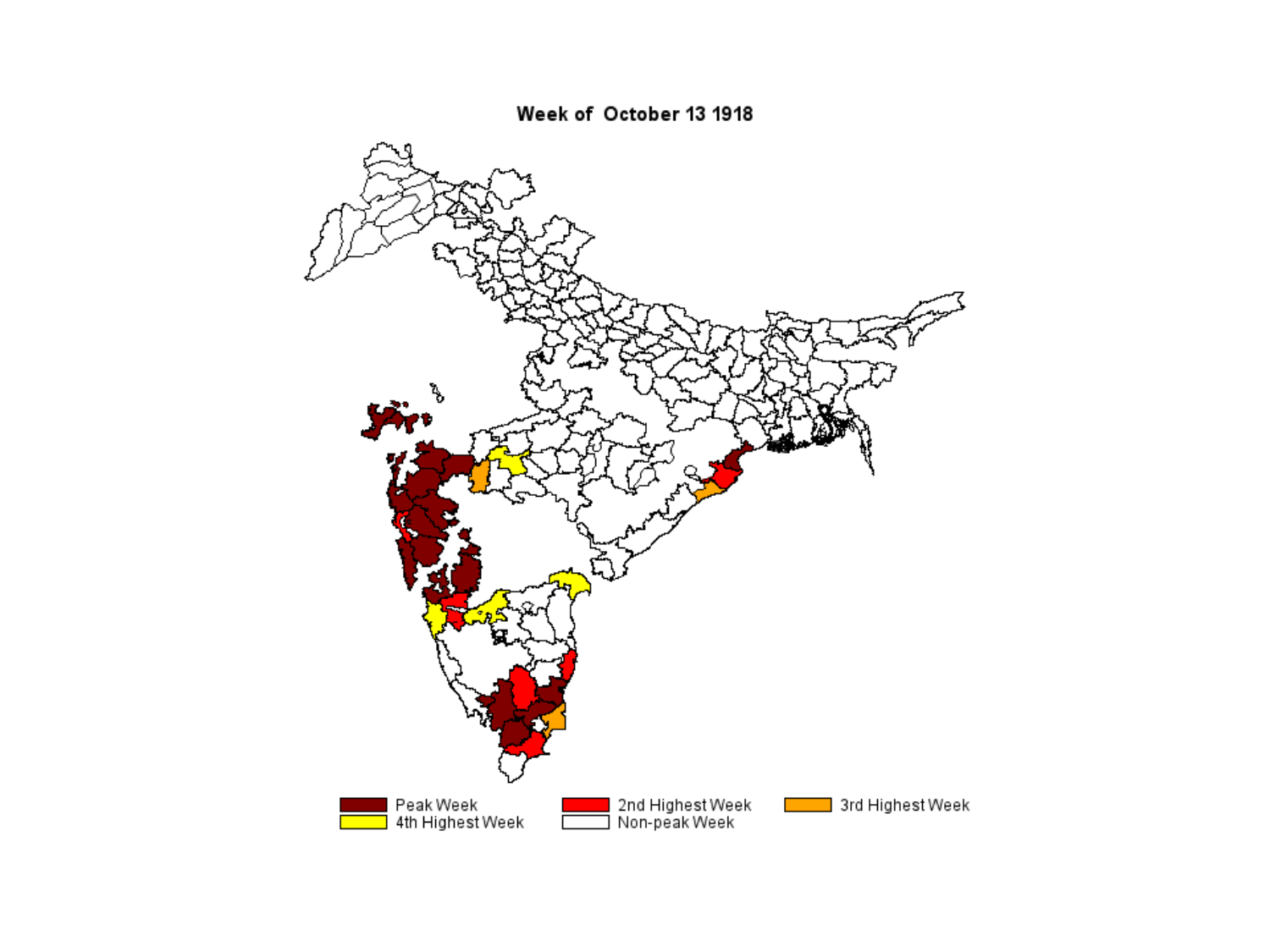

## Slide 9
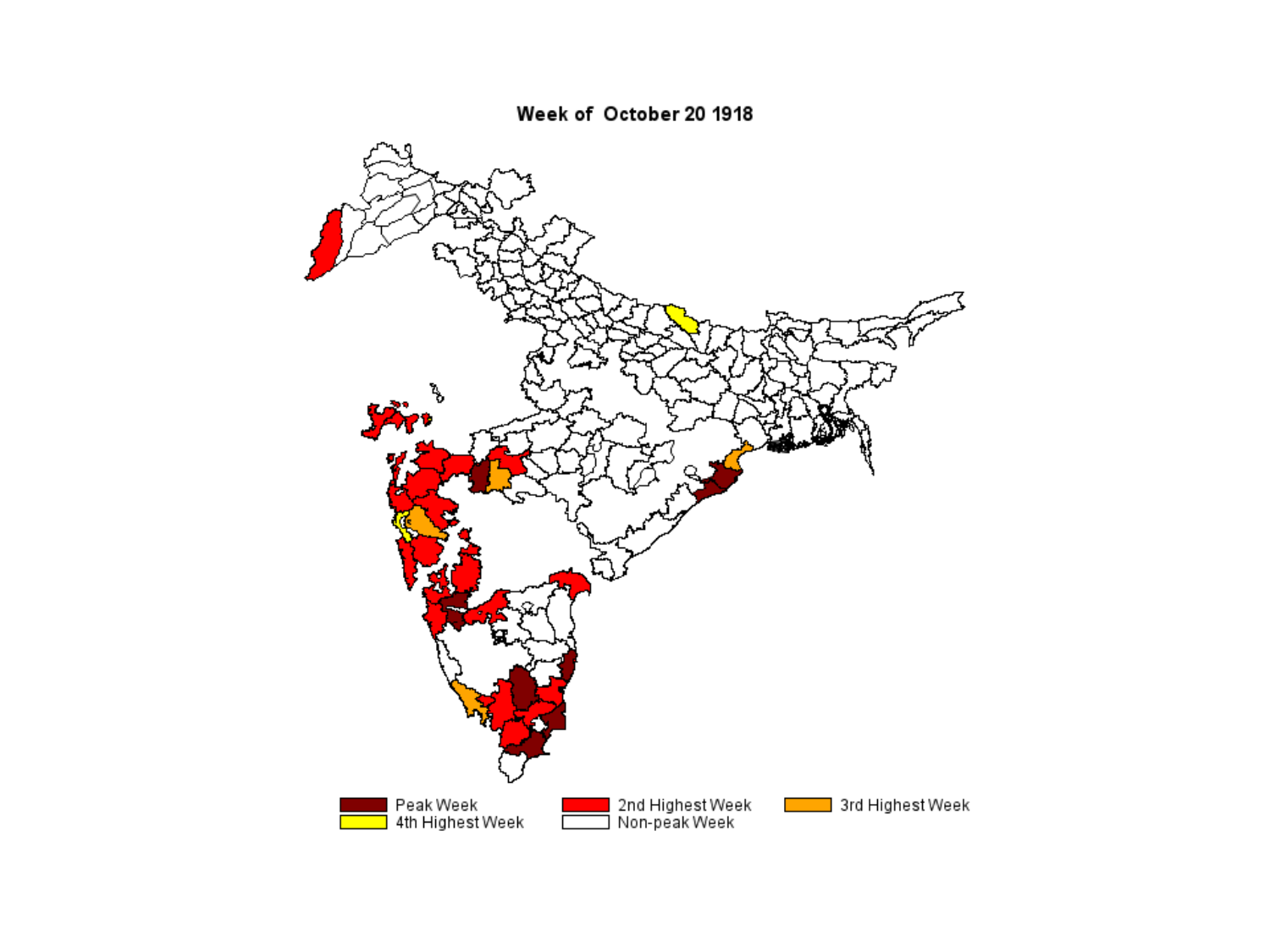

## Slide 10
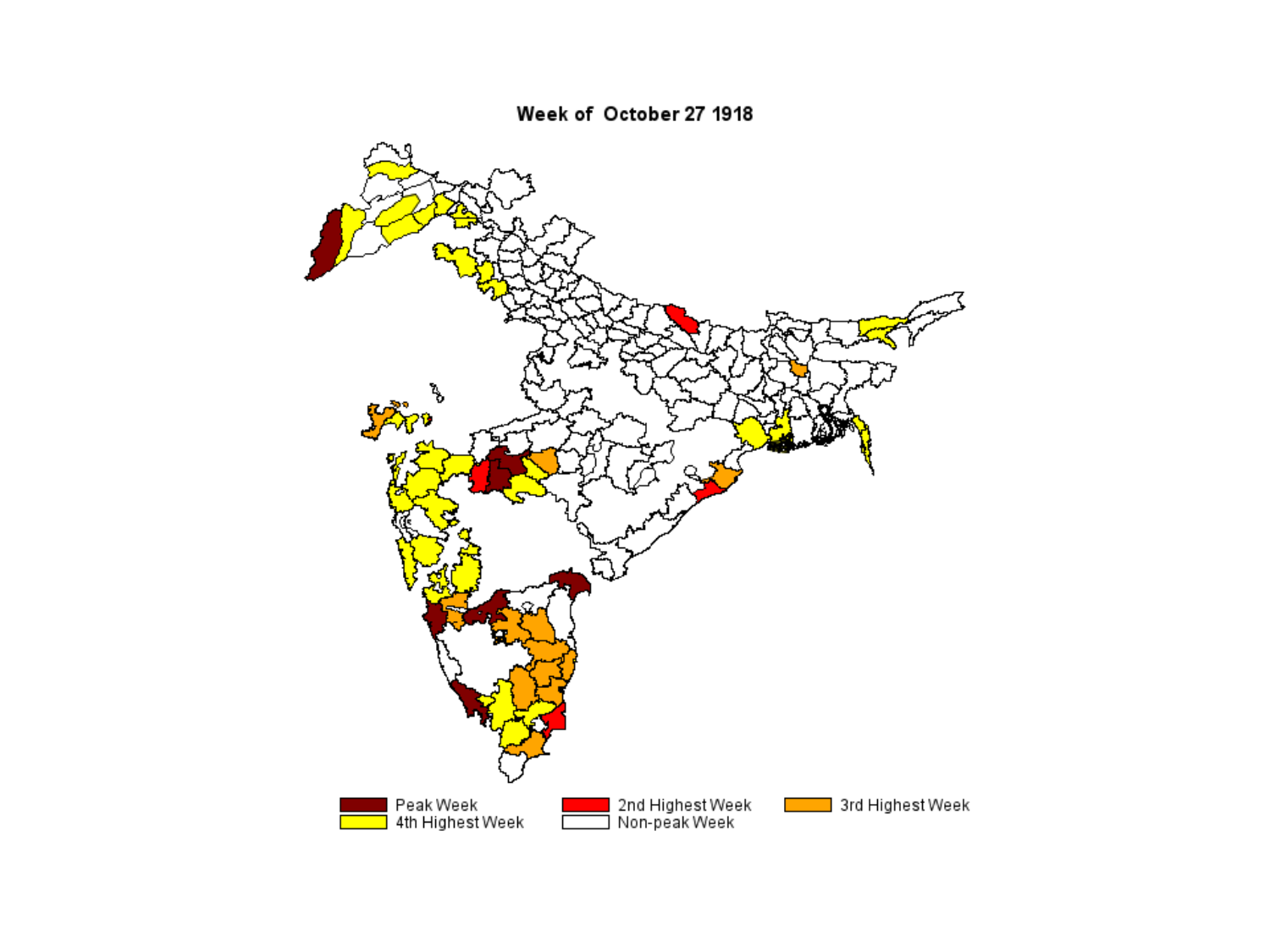

## Slide 11
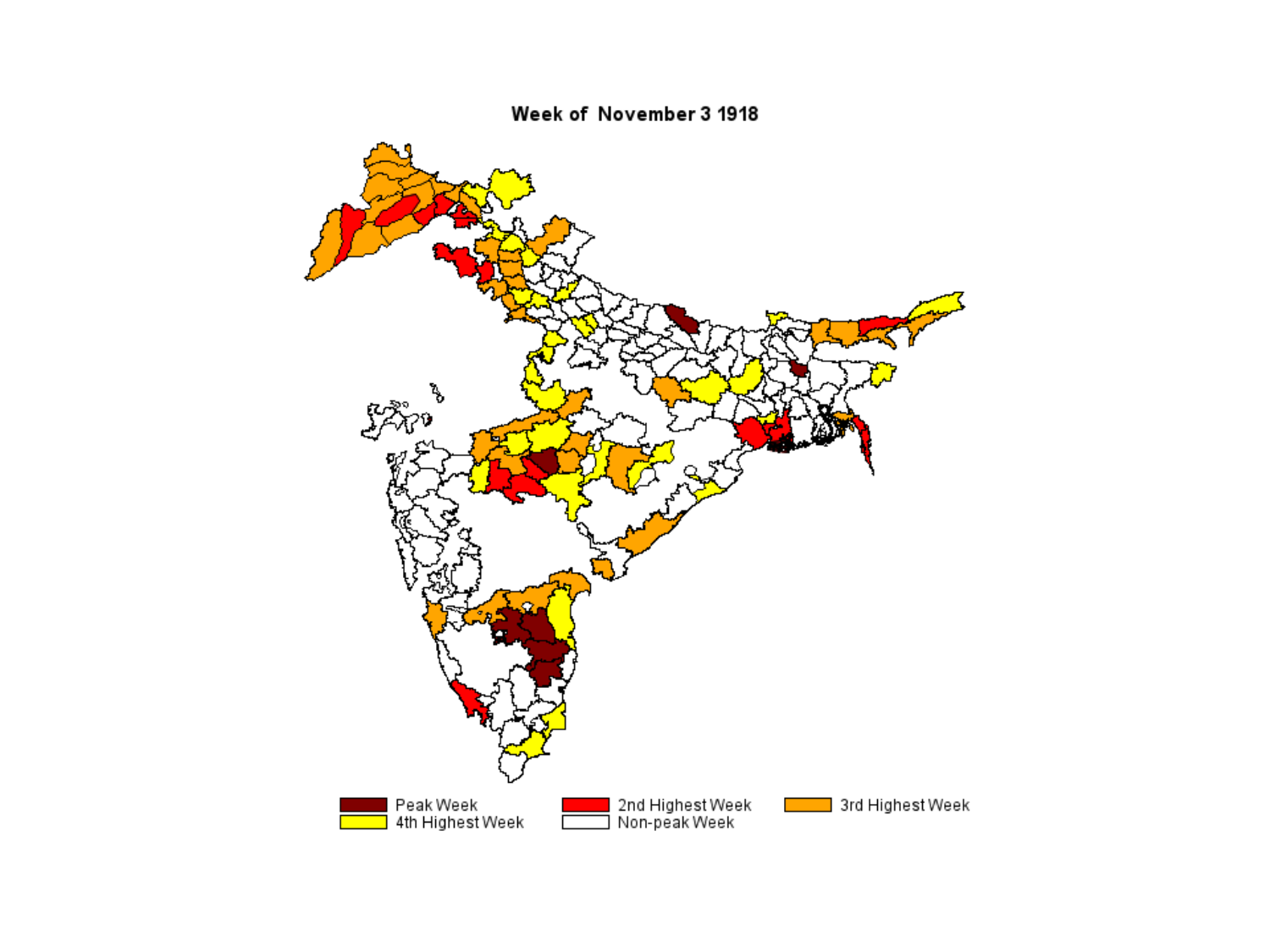

## Slide 12
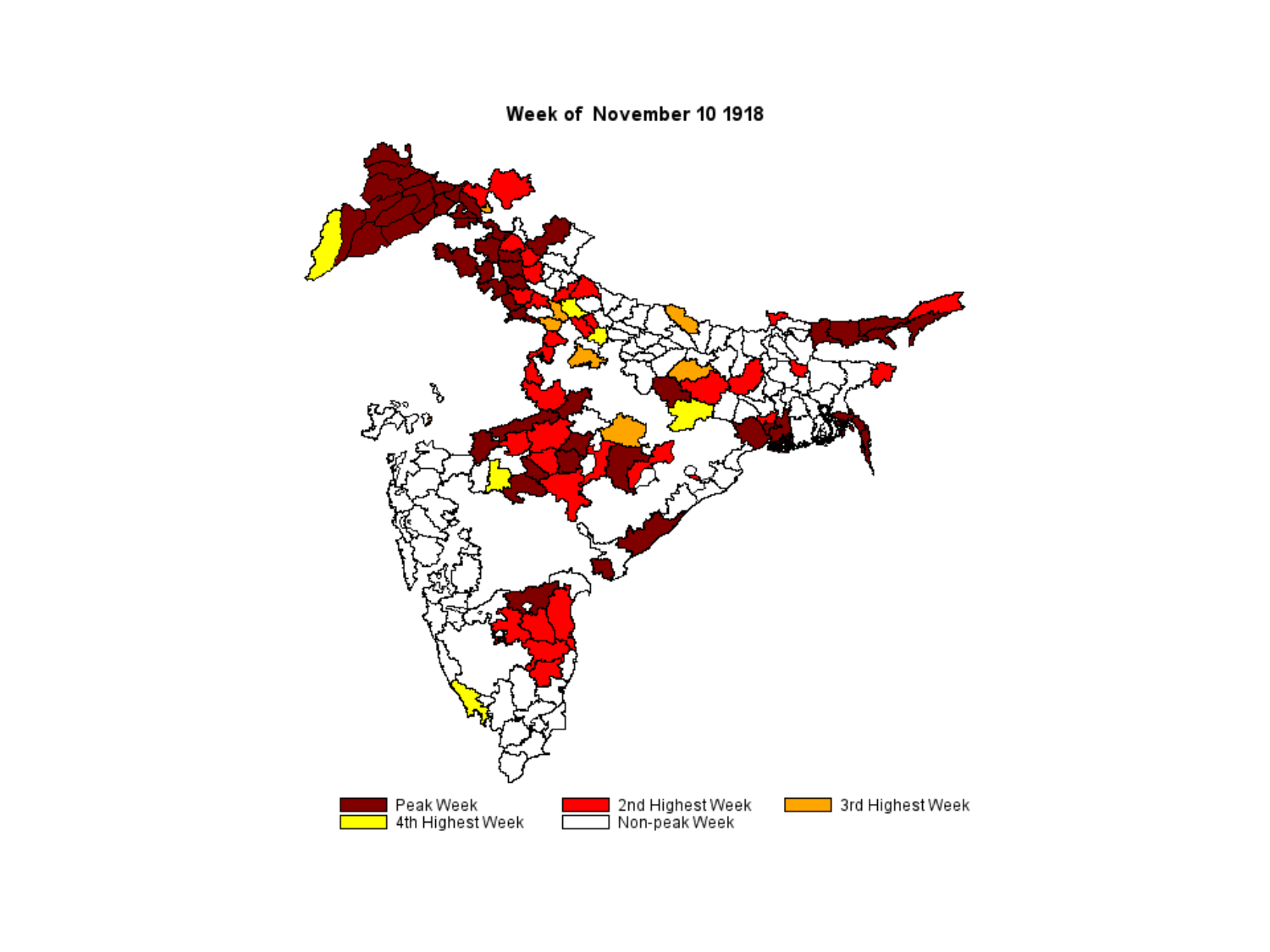

## Slide 13
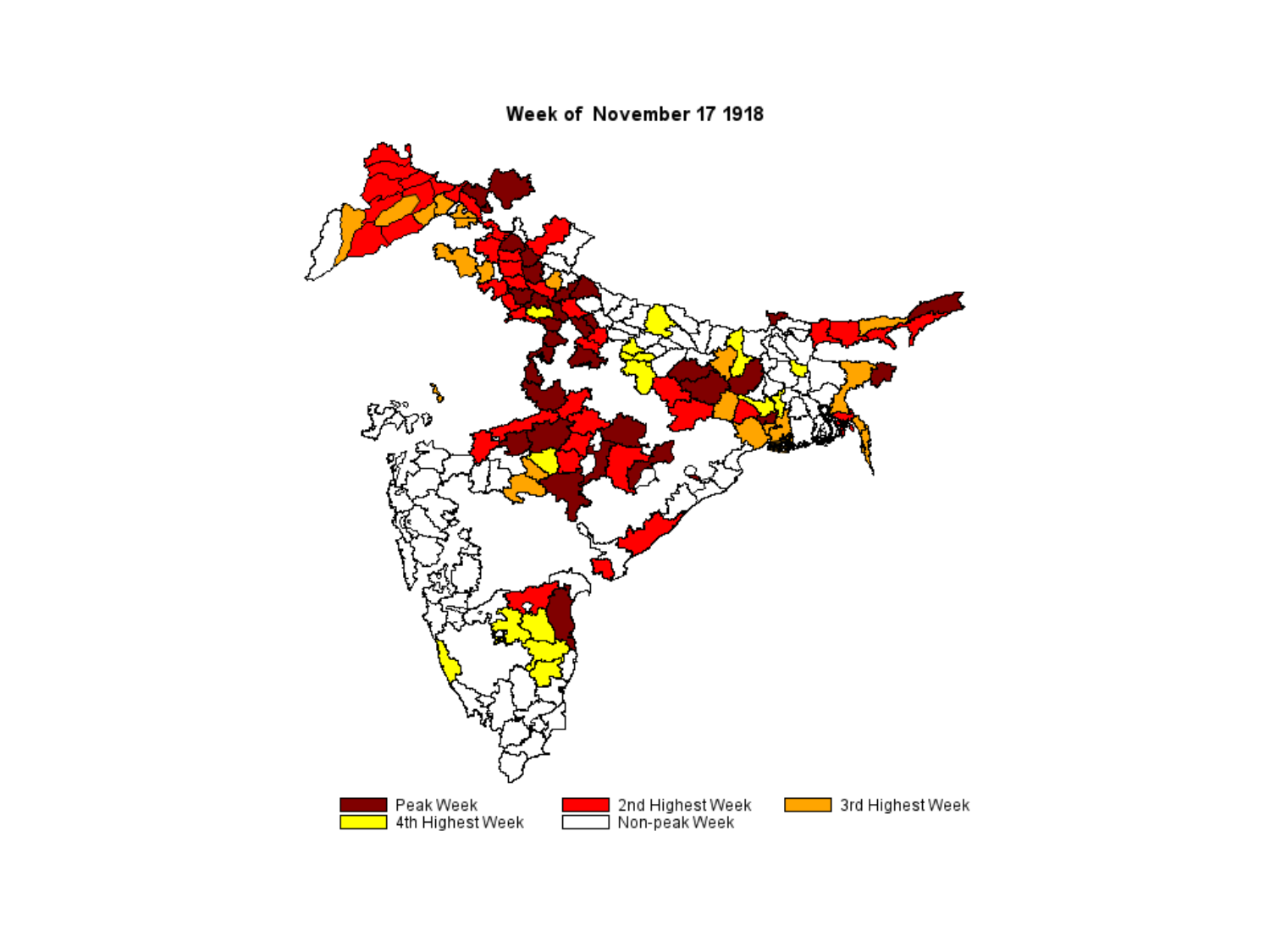

## Slide 14
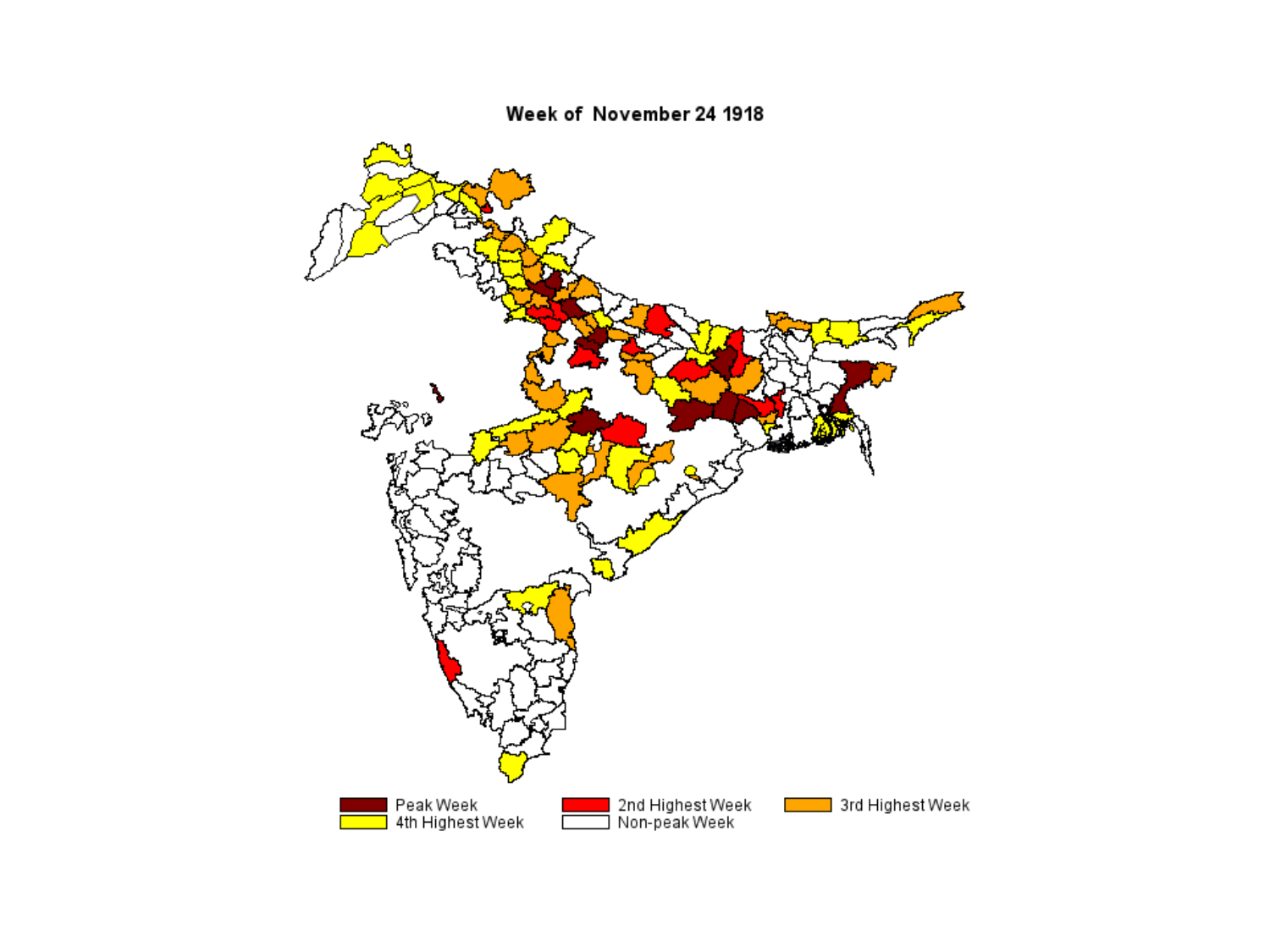

## Slide 15
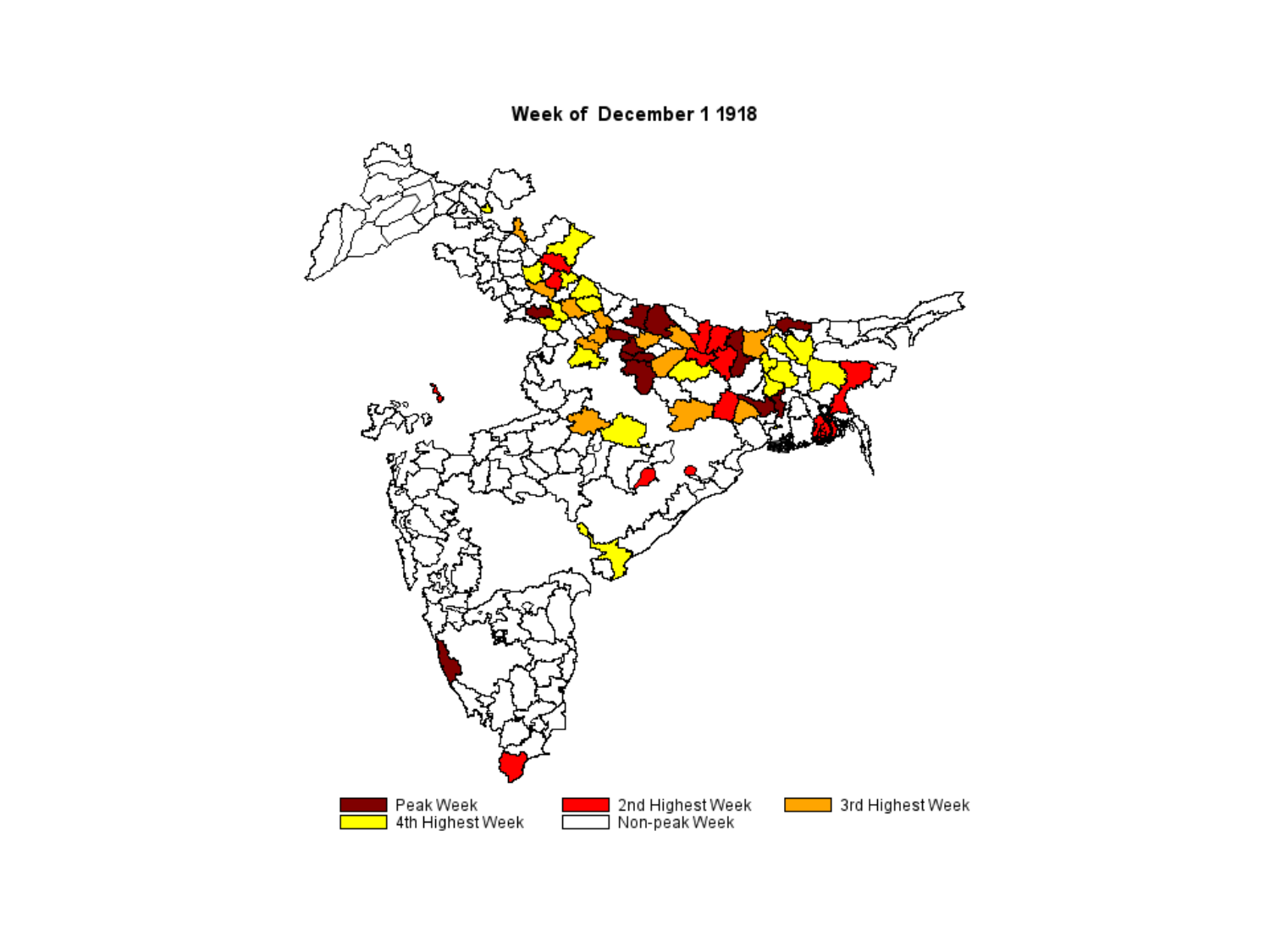

## Slide 16
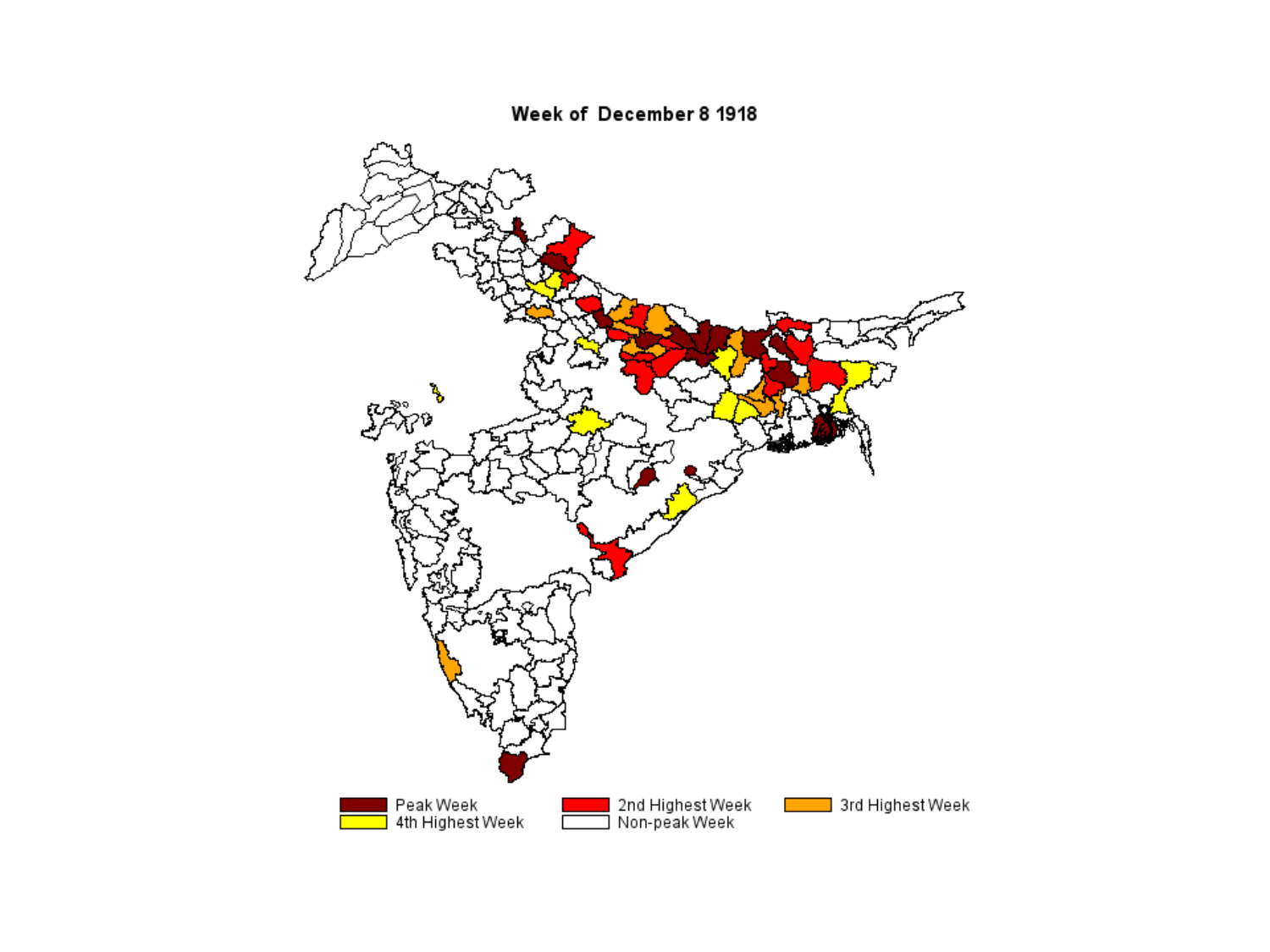

## Slide 17
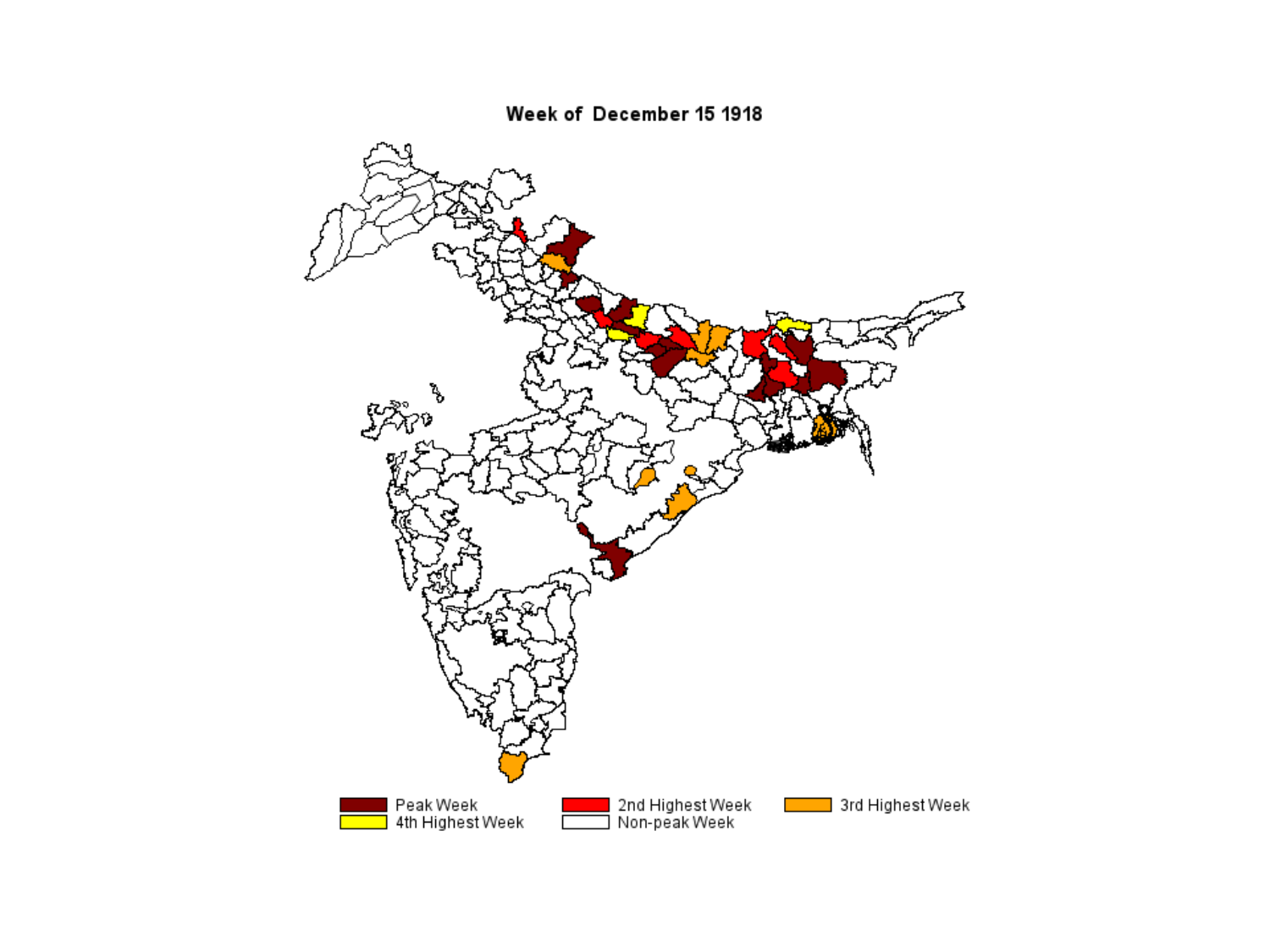

## Slide 18
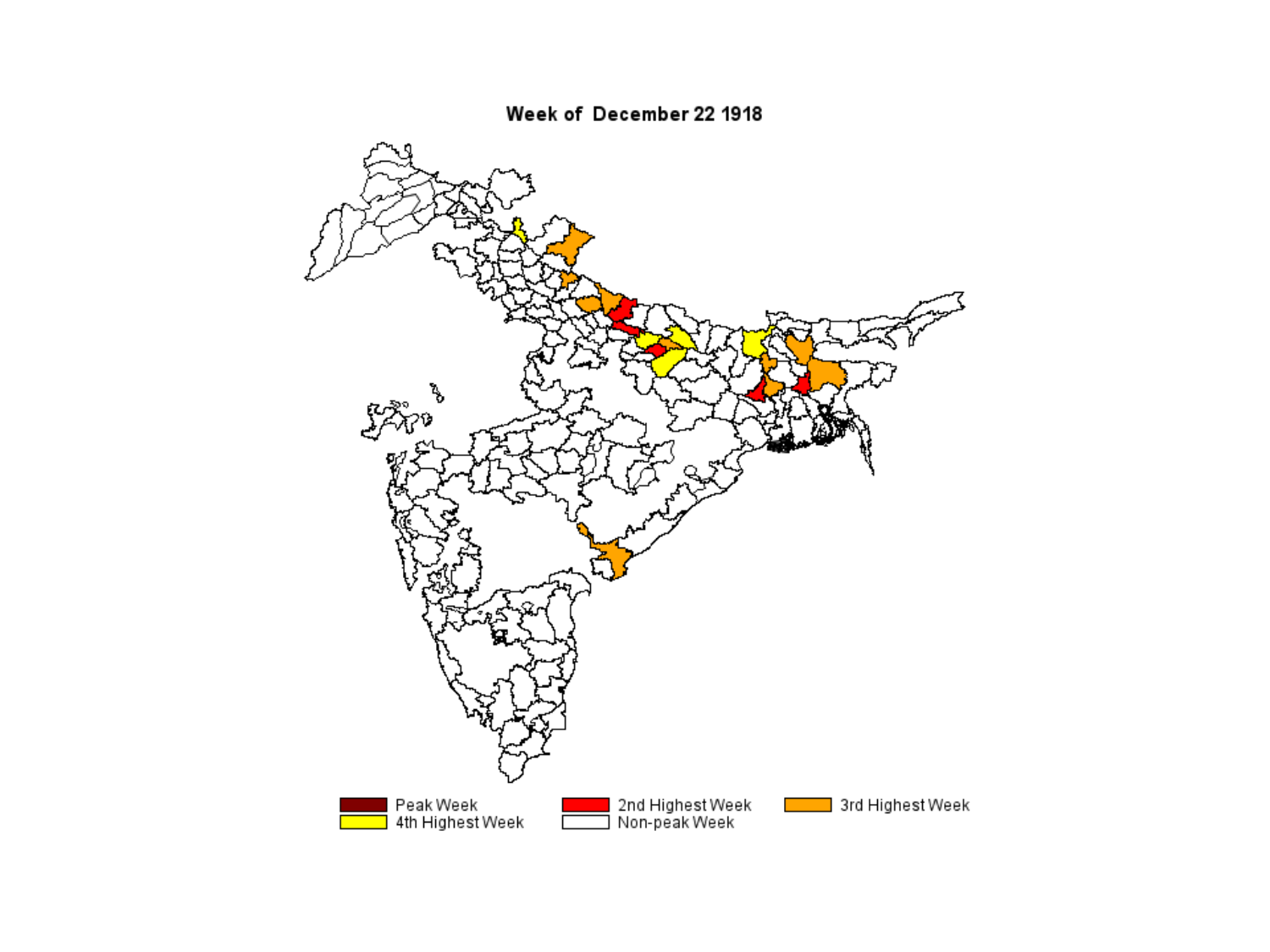

## Slide 19
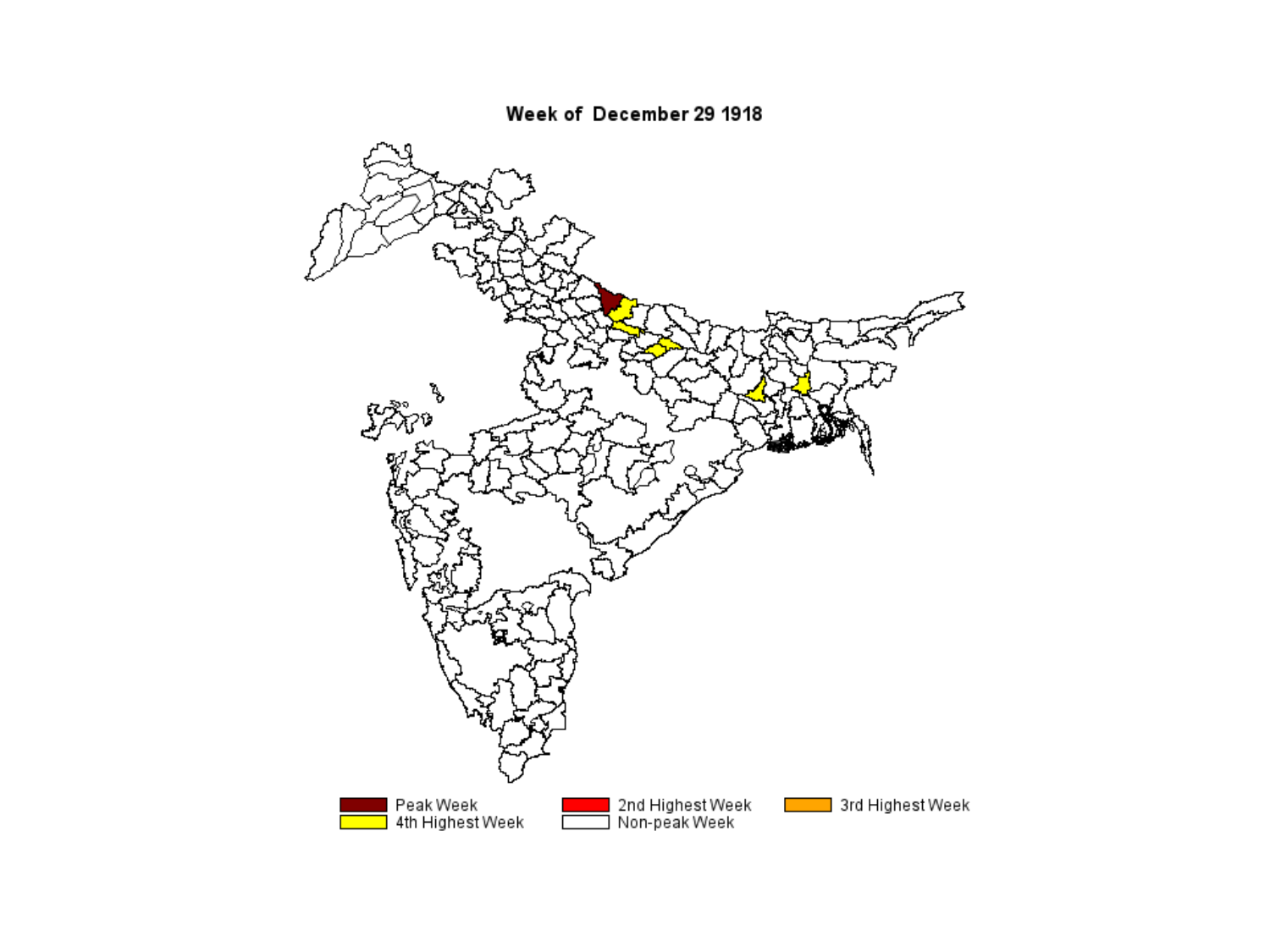

## Slide 20
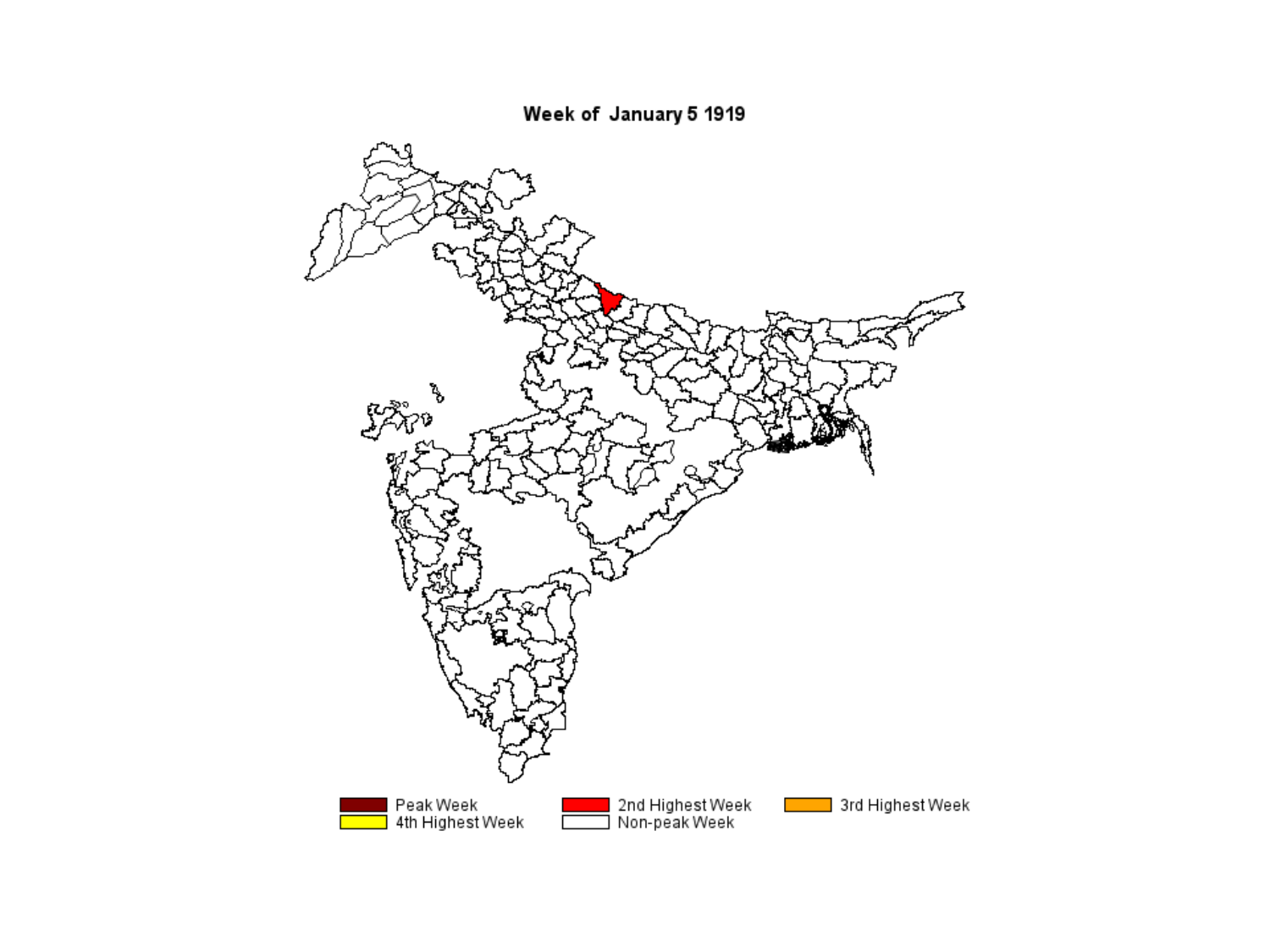

## Slide 21
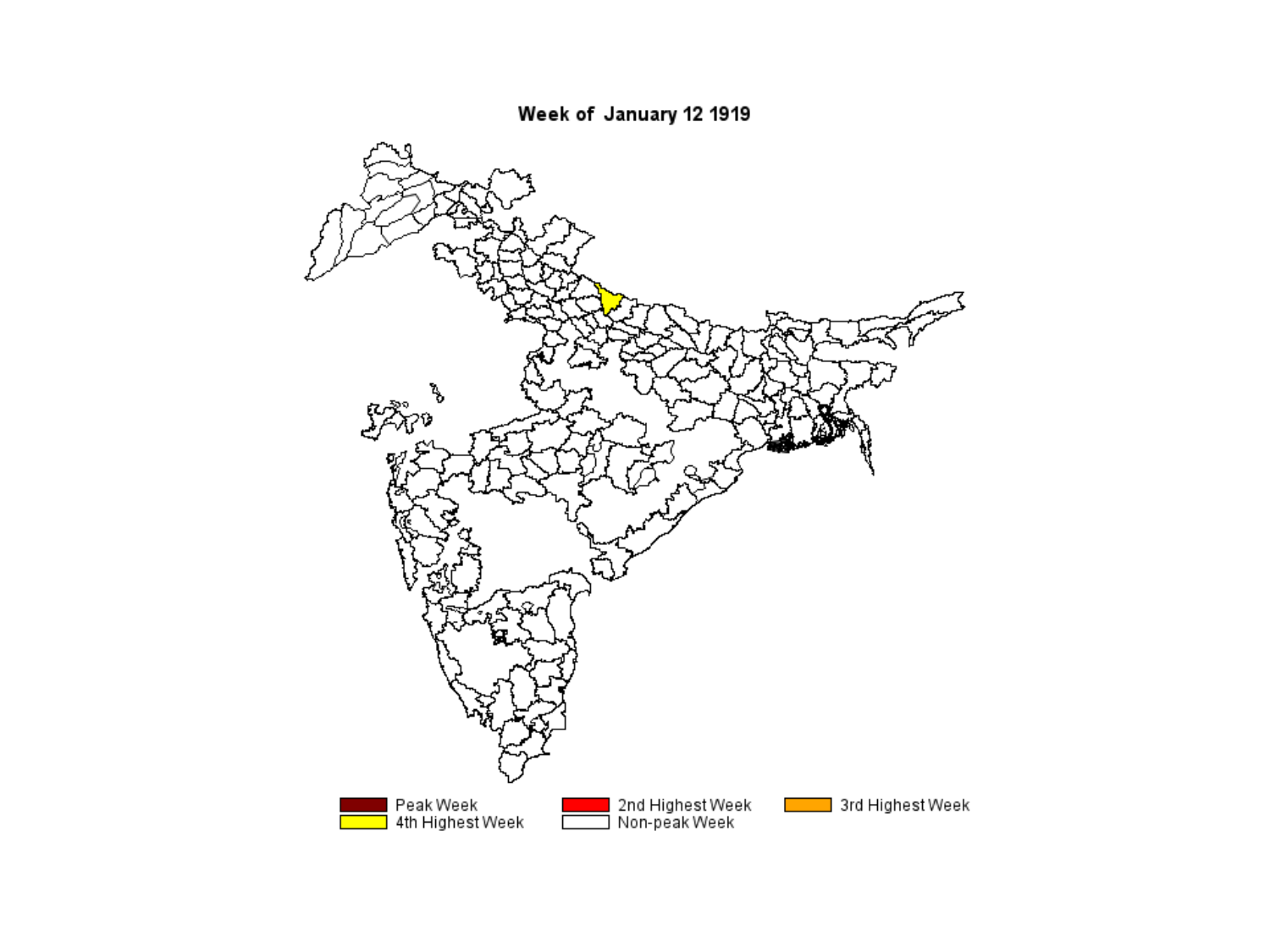

## Slide 22
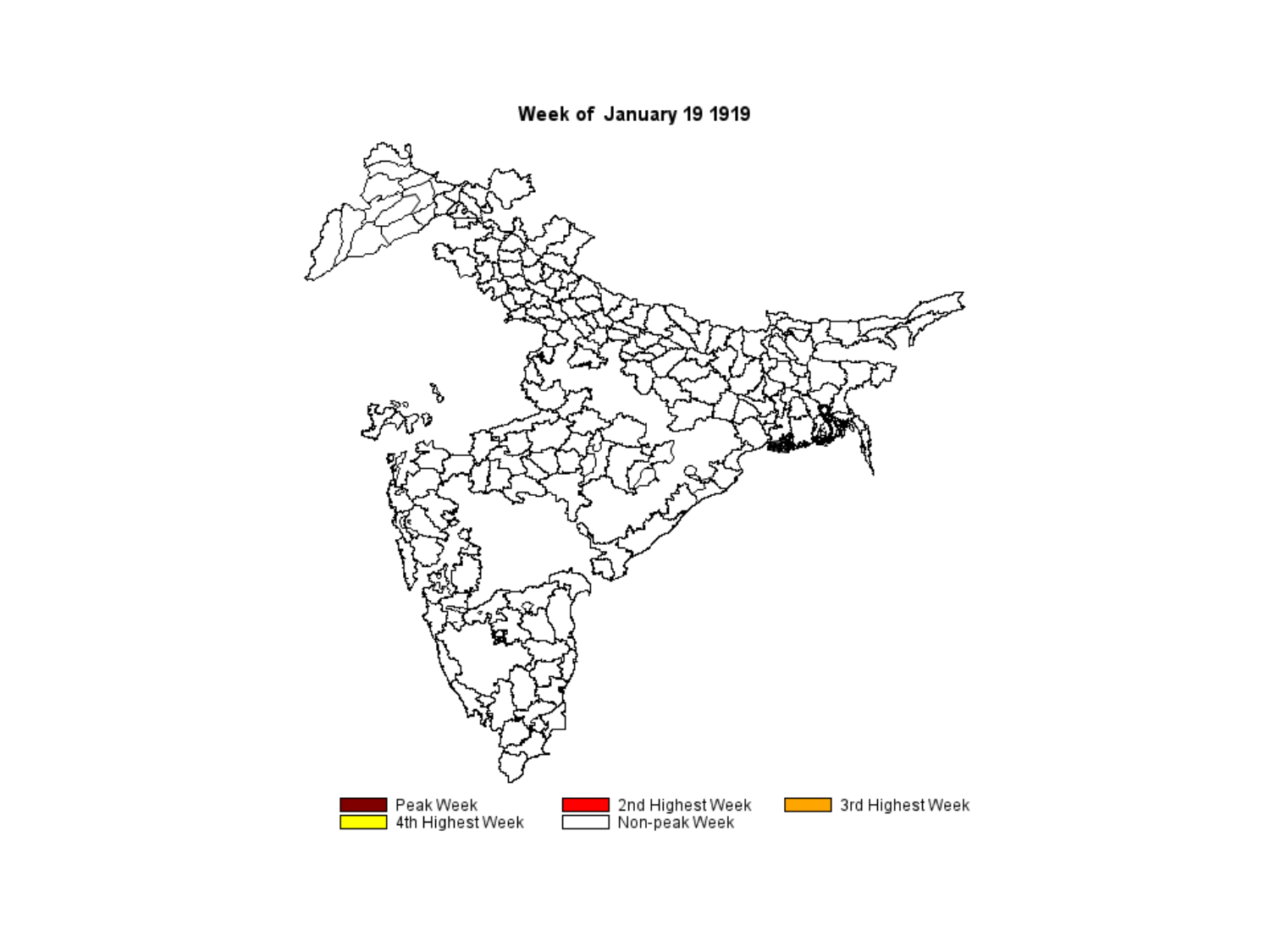

## Slide 23
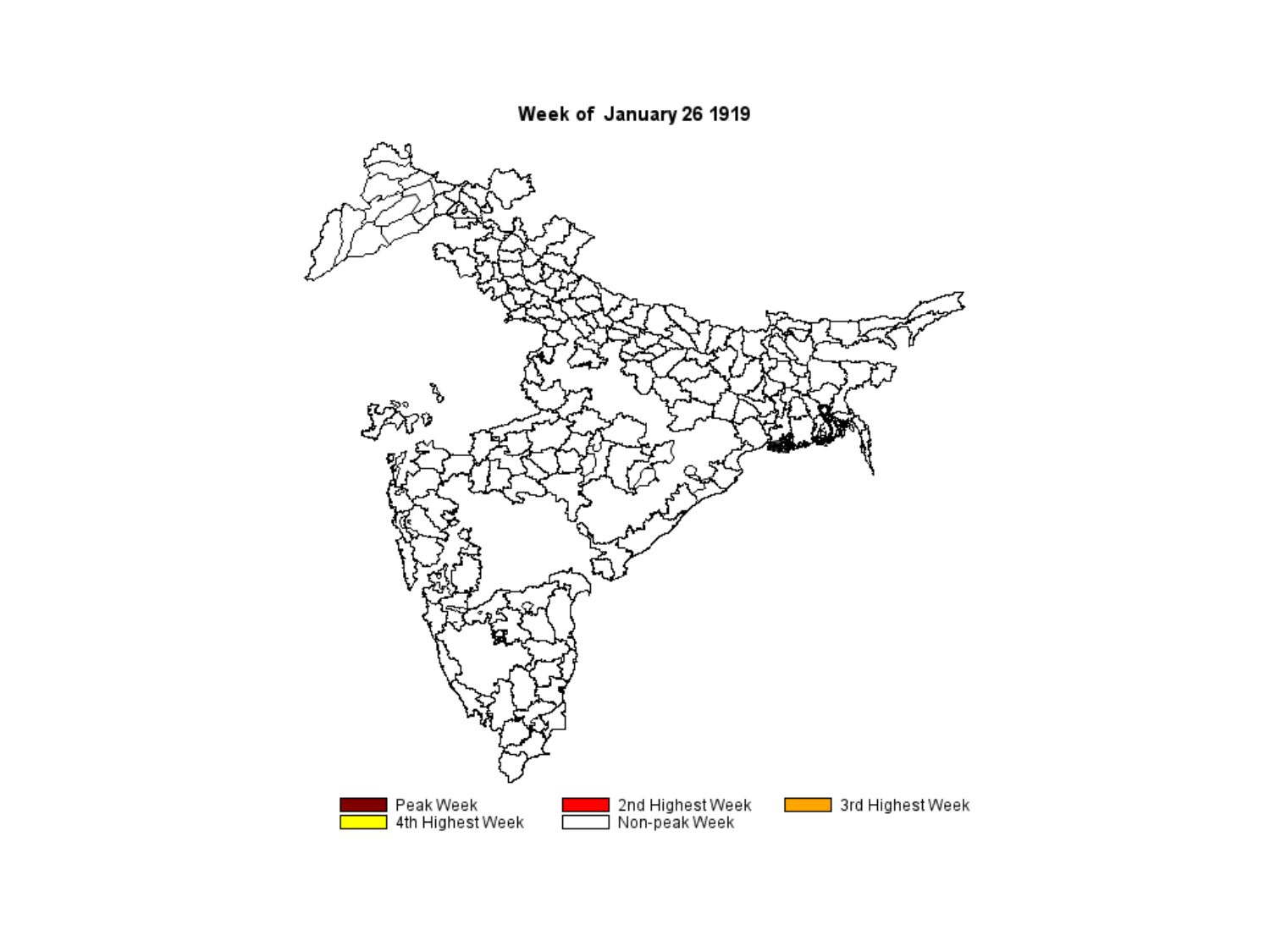

## Slide 24
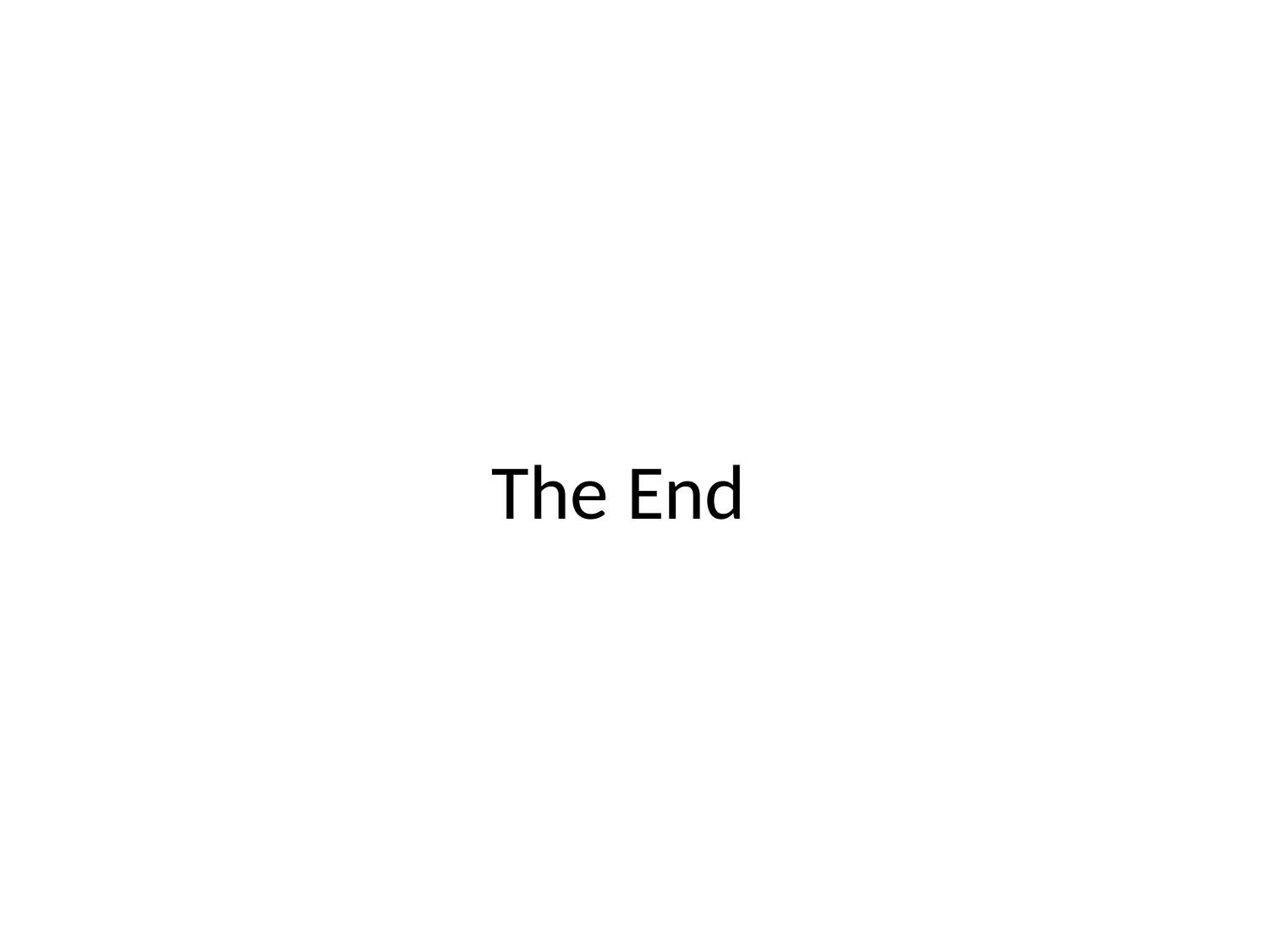

The End
