# Supplementary figures and images for: The evolution of pandemic influenza: evidence from India, 1918–19
Source: BMC Infect Dis. 2014 Sep 19;14:510. doi: 10.1186/1471-2334-14-510 (PMC4262128; doi:10.1186/1471-2334-14-510)

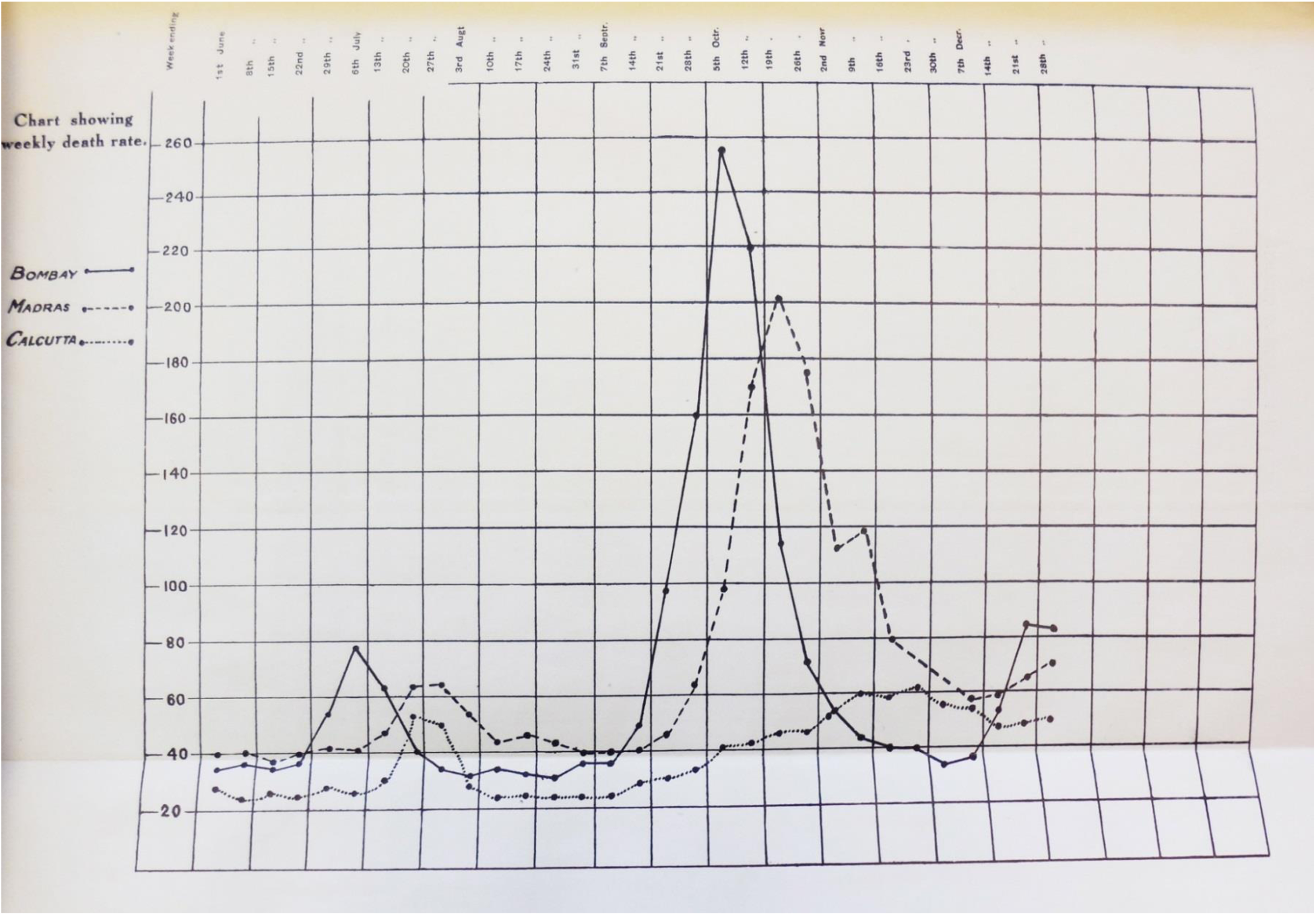

Supplement: Supplementary file 2 — Authors’ original file for figure 1 [file 12879_2014_3837_MOESM2_ESM.tiff]

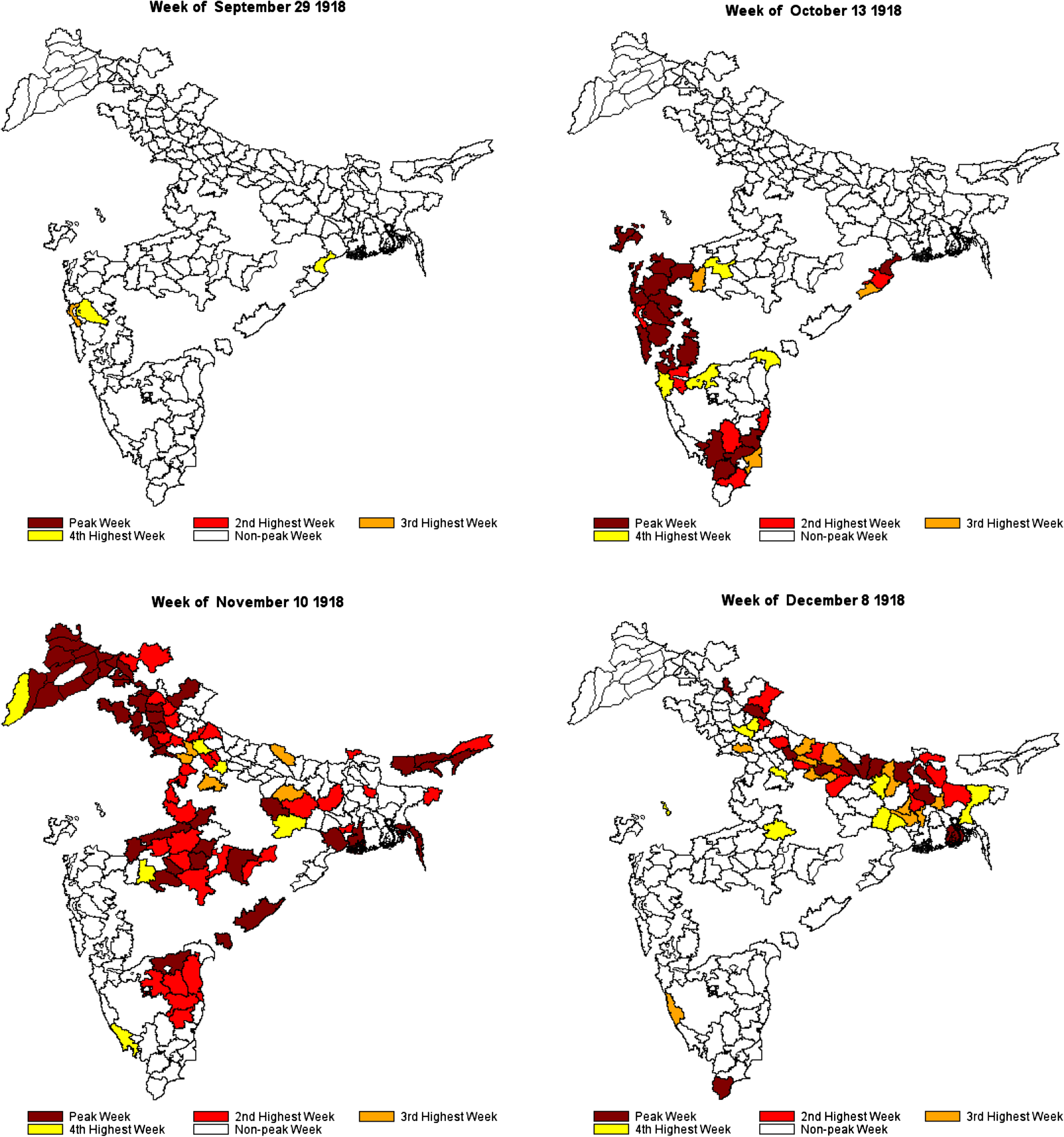

Supplement: Supplementary file 3 — Authors’ original file for figure 2 [file 12879_2014_3837_MOESM3_ESM.tiff]

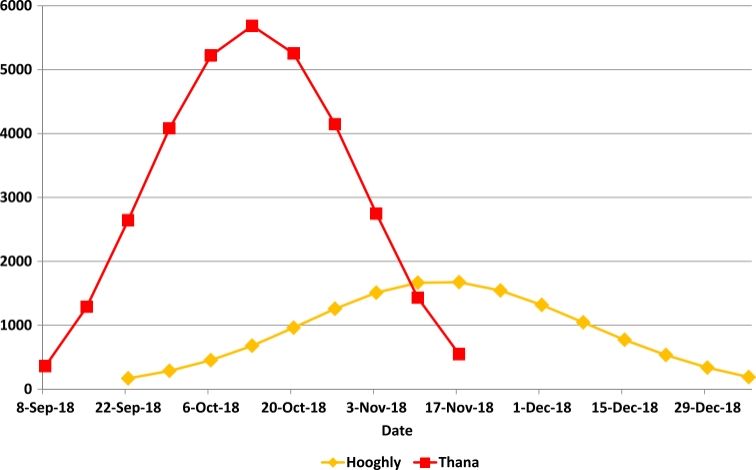

Supplement: Supplementary file 4 — Authors’ original file for figure 3 [file 12879_2014_3837_MOESM4_ESM.pdf]

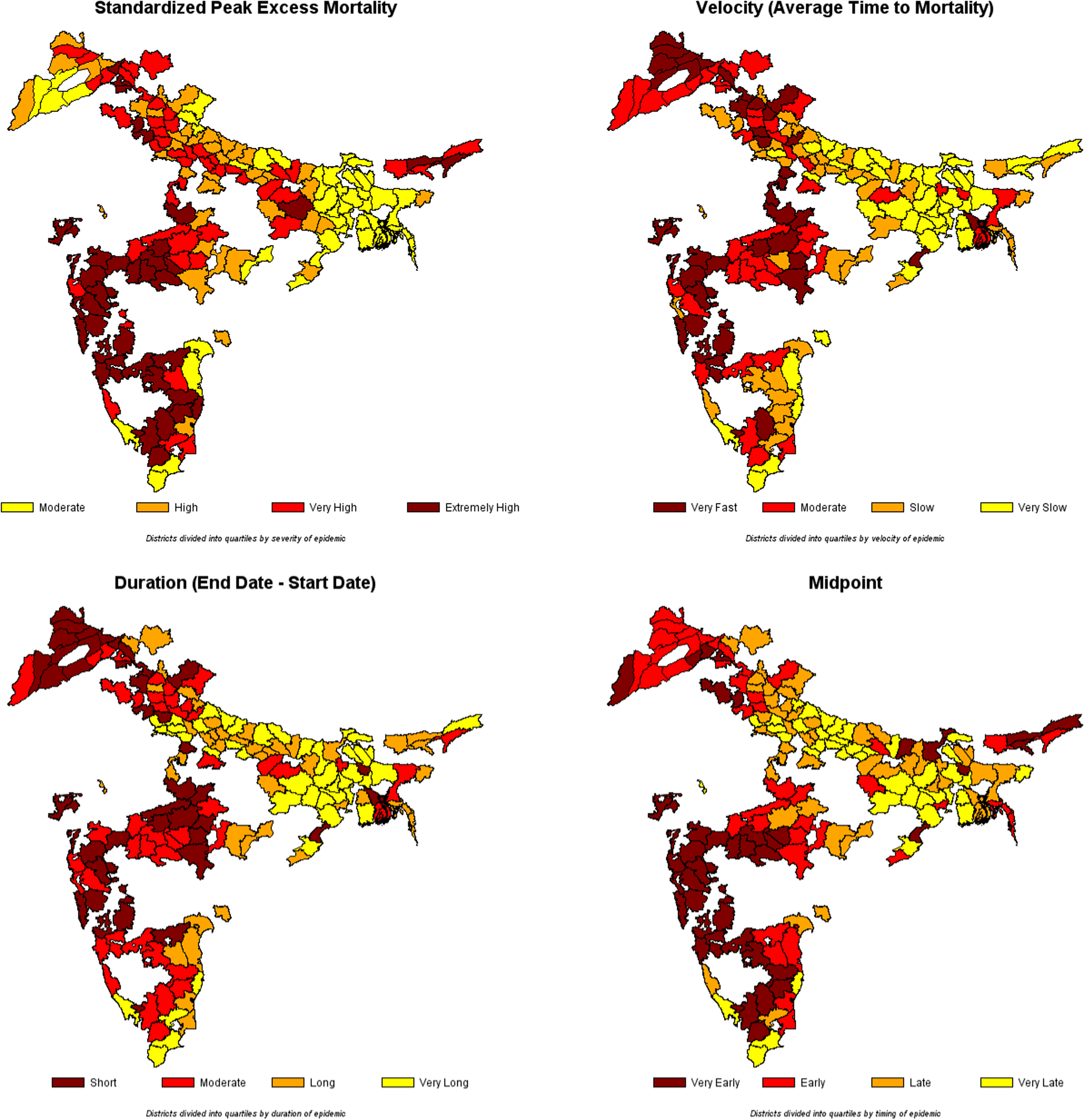

Supplement: Supplementary file 6 — Authors’ original file for figure 5 [file 12879_2014_3837_MOESM6_ESM.tiff]

**Bombay**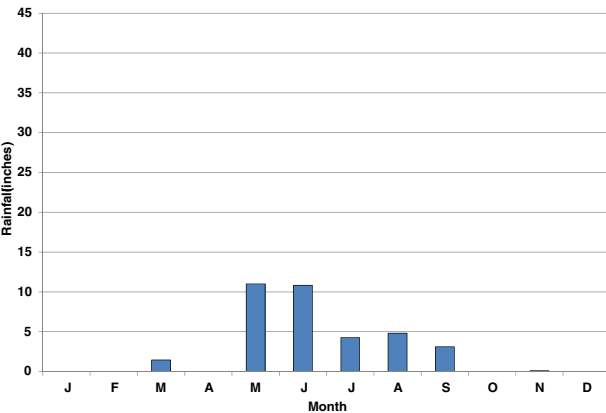**Calcutta**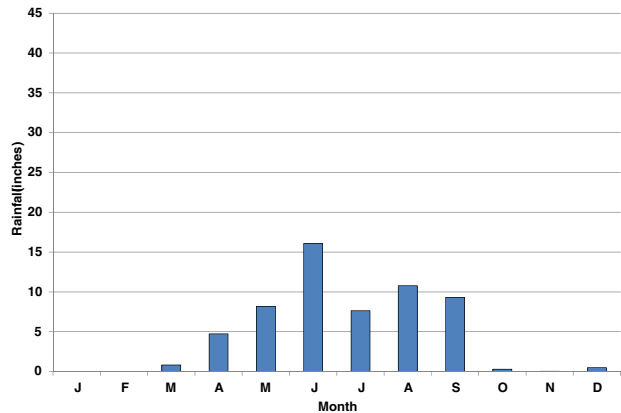**Madras**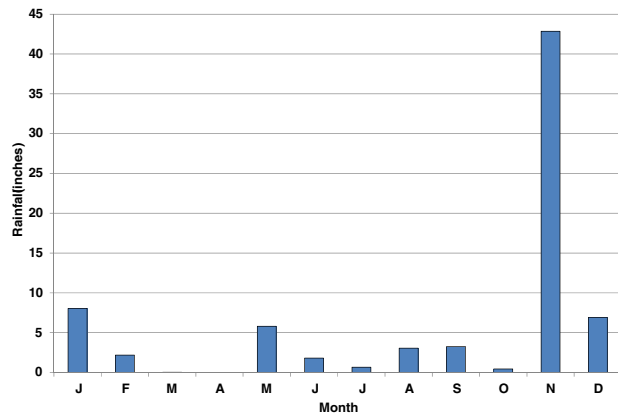

Supplement: Supplementary file 7 — Authors’ original file for figure 6 [file 12879_2014_3837_MOESM7_ESM.pdf]
