# Supplementary material for: The evolution of pandemic influenza: evidence from India, 1918–19
Source: BMC Infect Dis. 2014 Sep 19;14:510. doi: 10.1186/1471-2334-14-510 (PMC4262128; doi:10.1186/1471-2334-14-510)
Supplement: Supplementary file 5 — Authors’ original file for figure 4 [file 12879_2014_3837_MOESM5_ESM.pdf]

		SEVERITY OF WAVE		LENGTH OF WAVE		TIMING OF WAVE		SHAPE OF WAVE	
		Peak Excess Mortality	Standardized Peak Excess Mortality	Mean Time to Mortality	Duration of Wave	Peak Date of Wave	Midpoint of Wave	Variance of Wave	Wave Symmetry
SEVERITY OF WAVE	Peak Excess Mortality	1.00							
	Standardized Peak Excess Mortality	0.61 *<.0001*	1.00						
LENGTH OF WAVE	Mean Time to Mortality	-0.37 *<.0001*	-0.39 *<.0001*	1.00					
	Duration of Wave	-0.33 *<.0001*	-0.36 *<.0001*	0.94 *<.0001*	1.00				
TIMING OF WAVE	Peak Date of Wave	-0.24 *0.00*	-0.38 *<.0001*	0.36 *<.0001*	0.31 *<.0001*	1.00			
	Midpoint of Wave	-0.27 *<.0001*	-0.39 *<.0001*	0.68 *<.0001*	0.78 *<.0001*	0.70 *<.0001*	1.00		
SHAPE OF WAVE	Variance of Wave	-0.22 *0.00*	-0.25 *0.00*	0.85 *<.0001*	0.88 *<.0001*	0.14 *0.05*	0.69 *<.0001*	1.00	
	Wave Symmetry	-0.03 *0.67*	-0.13 *0.06*	0.09 *0.20*	0.37 *<.0001*	0.05 *0.44*	0.44 *<.0001*	0.17 *0.01*	1.00

*Epidemic Definition: Mortality is 1σ above Baseline.

*Pearson Correlation Coefficients, N = 213.

**p*-values for null hypothesis of 0 correlation in italics. Shaded correlations are significant at the 1% level.
